# Paper-based microfluidic system for tear electrolyte analysis†
†We declare no competing financial interests.
‡
‡Electronic supplementary information (ESI) available: Microscopic images of G1 paper and G41 paper under brightfield; optimization of CO_2_ laser radiation fluence and beam speed for ablating filter paper-G1; photographs of DI water diffusion in microfluidic channels with different lengths, different widths, different viscosities of fluid and different numbers of channels; fluorescence intensity readouts of Na^+^ and K^+^ ions with varied concentrations of fluorescent probes; effect of variations in temperature on fluorescence intensity; photographs of DMSO on G1 paper dried in the air; calibration curves of electrolyte sensing on G1 paper using microplate reader measurement; calculation of sensitivity of the fluorescent sensors based on International Union of Pure and Applied Chemistry (IUPAC) guidelines; quantification of ion interference in buffer solution and artificial tear fluid; light attenuation of LED lights using different optical filters; the design of the sample collection device and its potential clinical use; calibration curves of electrolyte sensors using the paper-based microfluidic system; quantifications of evaporation effect on sampling process; design of the sample collection device and its potential clinical use; batch-to-batch variation experiments; equation for background subtraction; movies of sample collection and measurements. See DOI: 10.1039/c6lc01450j



**DOI:** 10.1039/c6lc01450j

**Published:** 2017-02-16

**Authors:** Ali K. Yetisen, Nan Jiang, Ali Tamayol, Guillermo U. Ruiz-Esparza, Yu Shrike Zhang, Sofía Medina-Pando, Aditi Gupta, James S. Wolffsohn, Haider Butt, Ali Khademhosseini, Seok-Hyun Yun

**Affiliations:** a Biomaterials Innovation Research Center , Division of Biomedical Engineering , Brigham and Women's Hospital , Harvard Medical School , Cambridge , Massachusetts 02139 , USA . Email: akyetisen@gmail.com; b Harvard-MIT Division of Health Sciences and Technology , Massachusetts Institute of Technology , Cambridge , Massachusetts 02139 , USA . Email: syun@mgh.harvard.edu; c State Key Laboratory of Advanced Technology for Materials Synthesis and Processing , Wuhan University of Technology , 122 Luoshi Road , Wuhan , 430070 , China; d Ophthalmic Research Group , School of Life and Health Sciences , Aston University , Aston Triangle , Birmingham B4 7ET , UK; e Nanotechnology Laboratory , School of Engineering , University of Birmingham , Birmingham B15 2TT , UK; f Department of Physics , King Abdulaziz University , Jeddah , 21589 , Saudi Arabia; g Department of Bioindustrial Technologies , College of Animal Bioscience and Technology , Konkuk University , Hwayang-dong , Gwangjin-gu , Seoul 143-701 , Republic of Korea; h Harvard Medical School and Wellman Center for Photomedicine , Massachusetts General Hospital , 65 Landsdowne Street , Cambridge , Massachusetts 02139 , USA

## Abstract

The analysis of tear constituents at point-of-care settings has a potential for early diagnosis of ocular disorders such as dry eye disease, low-cost screening, and surveillance of at-risk subjects. However, current minimally-invasive rapid tear analysis systems for point-of-care settings have been limited to assessment of osmolarity or inflammatory markers and cannot differentiate between dry eye subclassifications. Here, we demonstrate a portable microfluidic system that allows quantitative analysis of electrolytes in the tear fluid that is suited for point-of-care settings. The microfluidic system consists of a capillary tube for sample collection, a reservoir for sample dilution, and a paper-based microfluidic device for electrolyte analysis. The sensing regions are functionalized with fluorescent crown ethers, *o*-acetanisidide, and seminaphtorhodafluor that are sensitive to mono- and divalent electrolytes, and their fluorescence outputs are measured with a smartphone readout device. The measured sensitivity values of Na^+^, K^+^, Ca^2+^ ions and pH in artificial tear fluid were matched with the known ion concentrations within the physiological range. The microfluidic system was tested with samples having different ionic concentrations, demonstrating the feasibility for the detection of early-stage dry eye, differential diagnosis of dry eye sub-types, and their severity staging.

## Abstract 2

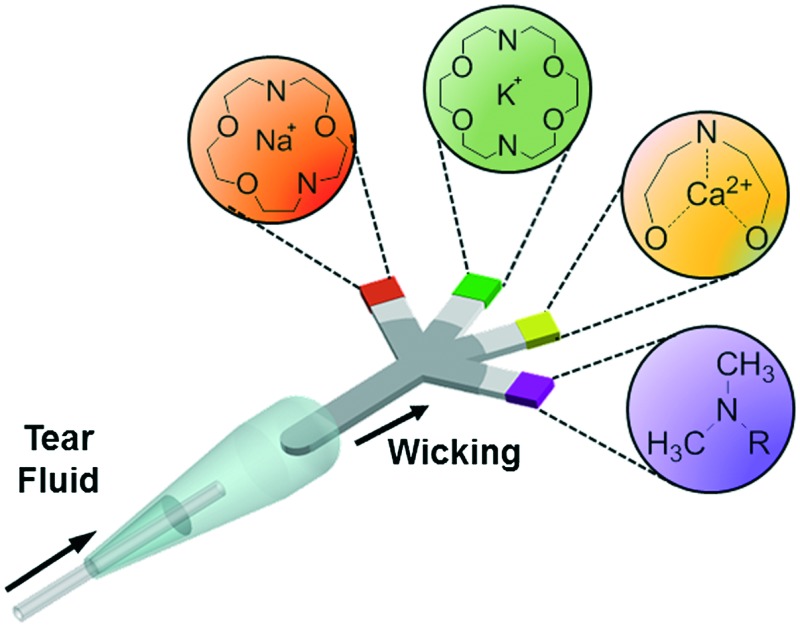
This article describes a paper-based microfluidic system that quantifies the concentrations of tear electrolytes using a smartphone-based reader.

The tear fluid offers a broad potential for sensing the physiological status and for the diagnosis of ocular diseases and metabolic dysfunction.^1–3^ The tear film maintains a smooth optical refracting surface and minimizes and prevents the risk of the eye infection through its flushing action and antimicrobial constituents.^4,5^ It comprises an outer lipid layer, an aqueous layer, and an inner mucin layer.^6,7^ The aqueous layer (∼4–50 μm) consists of proteins and electrolytes.^8^ Inadequate tear production or rapid tear film evaporation results in dry eye syndrome, which is caused by meibomian gland dysfunction (MGD, blockage of oil glands) and/or lacrimal gland dysfunction (LGD, aqueous tear deficiency).^9,10^ Differentiating MGD and LGD and their severity stages are important because they require different treatment approaches (*e.g.*, eye drops (aqueous/lipid), unclogging the glands, heat treatment, punctal plugs).^11,12^ Without early diagnosis and accurate treatment of ocular disease, dry eye results in impaired vision, discomfort, and eventually blindness.^13^ Furthermore, the diagnosis of early-stage dry eye is required before and after refractive surgeries (*e.g.* laser-assisted *in situ* keratomileusis) that severs corneal nerves, often leading to post-operation dry eye.^14–16^ Additionally, dry eye is a reason for contact lens discontinuations. Early diagnosis can assist eye care practitioners to choose an appropriate lens type.^17,18^ However, quantitative diagnostics are required not only for the early detection of dry eye, but also differential diagnosis of its subtypes and severity stages.

The development of diagnostic devices for dry eye syndrome dates back to the 1900s. Otto Schirmer developed a semi-quantitative test for measuring tear volume on the ocular surface.^19^ Schirmer's test can be used without anesthetics for the measurement of reflex tear secretion in response to conjunctival stimulation.^19^ The pH-sensitive phenol red thread test is another semi-quantitative measurement device for detecting dry eye syndrome.^20^ The development of rapid diagnostic devices for analyzing tear fluid has been limited over the last two decades. An important development was a clinically-used benchtop osmometer (TearLab, San Diego, CA, USA).^21^ This device measures the conductivity in tear fluid (50 μL) and correlates the measurement with an osmolarity value, providing a quantitative readout to diagnose dry eye syndrome. However, clinical interpretation of osmolarity readings for the diagnosis of dry eye has been questioned.^22^ Lateral-flow assays have been also utilized for detecting analytes in tear fluid. In 2013, the Food and Drug Administration (FDA) approved a lateral-flow diagnostic test (InflammaDry, Rapid Pathogen Screening Inc., Sarasota, FL, USA) that measures the concentration of matrix metalloproteinase-9 (MMP-9) for the diagnosis of dry eye.^23,24^ The concentration of MMP-9 has been shown to be elevated in the tears of patients with dry eye disease.^25^ However, this assay requires multiple sample processing steps and gives a binary response limiting its value in determining severity. Additionally, Tearscan (Advanced Tear Diagnostics, Birmingham, AL, USA) is a lateral-flow assay that has a dynamic range from 0.25–2.50 mg mL^–1^ of lactoferrin which is an indicator of aqueous tear production.^26^ This test is potentially useful when combined with IgE (allergen testing) measurement.^27^ Recently, an alkaline microfluidic homogeneous immunoassay was demonstrated for the determination of the low-volume (<1 μL of tear) lactoferrin at clinically relevant concentrations.^28^ Another recent study utilized an inkjet-printed device for the measurement of lactoferrin concentration in tear fluid.^29^

The measurement of tear electrolytes can be used to identify dry eye at different severity stages and differentiate its sub-types such as MGD and LGD. The Na^+^ ion concentration in the tears of healthy individuals ranges between 120–165 mmol L^–1^.^30^ However, in dry eye syndrome caused by MGD or LGD, Na^+^ ion concentration in human tear increase was reported to be significant, which can be detected by a sensor sensitivity of ∼3.0 mmol L^–1^.^31^ Additionally, divalent metal ion concentration was found to be different in MGD and LGD. As compared to MGD, human tear Ca^2+^ ion concentration in LGD significantly increases and could be potentially detected by a sensor sensitivity of 20–40 μmol L^–1^.^31^ Hence, the accurate measurement of tear electrolytes can provide quantitative data for dry eye diagnosis. Therefore, the development of a minimally-invasive diagnostic system for the quantitative analysis of tear electrolytes is highly desirable for the detection of dry eye in different severity stages and classification of its subtypes. Such a quantitative device can be used to screen for at-risk dry eye patients, enable the early detection of dry eye, and improve management approaches.

This article reports on the development of a paper-based microfluidic system for application in the quantitative analysis of electrolytes in tear fluid (Fig. 1). Upon introducing a low-volume sample (*e.g.*, tear fluid), the microfluidic device distributed the sample into sensing regions that were functionalized with fluorescent sensing agents. The microfluidic device was placed in a portable readout device that consisted of various LED illumination wavelengths for fluorescence excitation. A smartphone application (app) was utilized to capture the fluorescence assay images which were digitally processed to obtain concentration values for the quantitative analysis of electrolytes in artificial tear fluid.

**Fig. 1:**
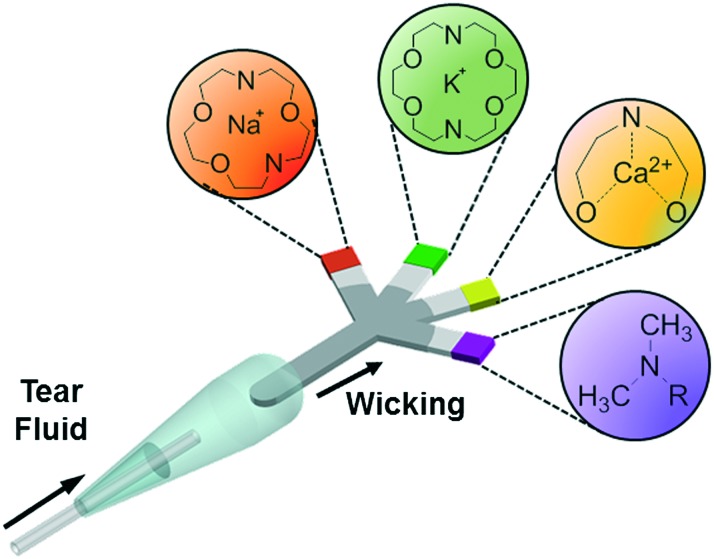
Principle of operation of the paper-based microfluidic system for the quantitative analysis of electrolytes in tear film.

## Experimental section

### Materials

All chemicals were of analytical grade and used without further purification. Tetra(tetramethylammonium) salt (tetrakis(*N*,*N*,*N*-trimethylmethanaminium)3,3′-{1,4,10-trioxa-7,13-diazacyclopentadecane-7,13-diylbis[(2,5-dimethoxy-4,1-phenylene)carbamoyl]}bis[6-(2,7-dichloro-6-oxido-3-oxo-3H-xanthen-9-yl)benzoate]) (fluorescent diaza-15-crown-5, HPLC purity ≥90%, *λ*_ex_/*λ*_em_: 507/532 nm); 1,3-benzenedicarboxylic acid, 4-[6-[16-[2-(2,4-dicarboxyphenyl)-5-methoxy-1-benzofuran-6-yl]-1,4,10,13-tetraoxa-7,16-diazacyclooctadec-7-yl]-5-methoxy-1-benzo] (fluorescent diaza-18-crown-6, HPLC purity ≥90%, *λ*_ex_/*λ*_em_: 346/500 nm); *N*-(2-methoxyphenyl)iminodiacetate chelator (*o*-acetanisidide, *λ*_ex_/*λ*_em_: 495/515 nm), and benzenedicarboxylic acid 2(or 4)-[10-(dimethylamino)-3-oxo-3H-benzo[*c*]xanthene-7-yl] (seminaphtorhodafluor, *λ*_ex_/*λ*_em_: 530/640 nm) were purchased from Thermo Fisher Scientific. Whatman qualitative filter paper (grade 1, grade 41), Tris base (99.9%), Tris hydrochloride (Tris HCl, 99%), sodium chloride (NaCl) (99%), potassium chloride (KCl) (99.0%), magnesium chloride (MgCl_2_) (98%), calcium chloride dihydrate (CaCl_2_·2H_2_O) (99%), iron(iii) chloride hexahydrate (FeCl_3_·6H_2_O) (97%), copper(ii) chloride dihydrate (CuCl_2_·2H_2_O) (99.0%), nickel(ii) sulfate hexahydrate (NiSO_4_·6H_2_O) (99%), ammonium chloride (NH_4_Cl) (99.0%), citric acid (99.0%), l-ascorbic acid (99.0%), albumin (bovine serum) (96%), lysozyme (chicken egg white) (90%), d-(+)-glucose (99.5%), dimethyl sulfoxide (DMSO)(anhydrous, 99.9%), Rhodamine B (95%) were purchased from Sigma-Aldrich. Polydimethylsiloxane (PDMS) elastomer (SYLGARD® 184 Silicone elastomer kit) was purchased from Dow Corning. An opaque black polymethyl methacrylate (PMMA) sheet (thickness: ∼3 mm) was purchased from McMaster-Carr.

### Equipment

A CO_2_ laser (VLS2.30) operating at a wavelength of 10.64 μm at 30 W was purchased from Universal Laser Systems. An optical microscope (IX51) with phase contrast and fluorescence imaging was purchased from Olympus. A charge-coupled device (CCD) (2 MP) color digital microscope camera was purchased from Spot RT3. A fluorescence plate reader (Synergy 2 Multi-Mode Reader) was purchased from BioTek. An electrochemical pH sensor was purchased from Mettler Toledo. Capillary tubes (5 μL) were purchased from CM Scientific. A digital single-lens reflex camera (D90, 12.3 MP) and a lens (AF-S DX 18–105 mm f/3.5–5.6G ED VR) were purchased from Nikon. A portable UV light (*λ* = 354 nm) was purchased from Thermo Scientific. Longpass colored glass filters were purchased from Thorlabs (420, 495, 515, 590 nm). SolidWorks (×64), CorelDRAW (×7), and ImageJ (1.50 h) were used for design and image processing. Images were captured using an iPhone 6S using a smartphone app (Shoot, ProCam). A charge-coupled device (CCD) spectrometer was purchased from Thorlabs (CCS100, 350–700 nm).

### Laser cutting of paper-based microfluidic devices

A CO_2_ laser (*λ* = 10.64 μm, 30 W) was used to pattern paper. Images were designed in CorelDRAW and defined as lines. The lengths of the paper-based microfluidic devices were varied from 10 to 40 mm with channel widths ranging from 2.0 to 5.0 mm. The fluence of laser beam was varied from 0 to 264 mJ mm^–2^ at beam speeds ranging from 30.0 to 65.5 mm s^–1^. The platform exhaust operated at 150 cfm and 6.0 mm in static pressure (255 m^3^ h^–1^, 1.5 kPa).

### Flow rate measurements

The paper-based microfluidic devices were fixed upright on a leveled surface. Sample solutions ranging from 10–20 μL were introduced from the inlet and images were taken with a digital single-lens reflex camera (12.3 MP) using a lens (AF-S DX 18–105 mm f/3.5–5.6G ED VR) operating at ISO1600, 1/1500 speed, and F3.5.

### Preparation of ion solutions, viscous samples, and artificial tear fluid

Two stock solutions containing Tris base and Tris HCl were mixed to obtain pH values from 4.5 to 9.0 with a constant ionic strength (150 mmol L^–1^) in deionized (DI) water (18.2 MΩ cm, Millipore) while the solution was monitored with an electrochemical pH sensor. NaCl, KCl, MgCl_2_, CaCl_2_·2H_2_O, FeCl_3_·6H_2_O, CuCl_2_·2H_2_O and NiSO_4_·6H_2_O were used to obtain ion concentrations ranging from 25 to 200 mmol L^–1^. d-(+)-glucose solutions (0.55–2.78 mol L^–1^) in DI water were prepared to obtain viscosity values ranging from 1.0 to 10.0 mPa s. Artificial tear fluid contained NaCl (125 mmol L^–1^), KCl (20 mmol L^–1^), CaCl_2_ (1 mmol L^–1^), MgCl_2_ (0.5 mmol L^–1^), urea (5 mmol L^–1^), NH_4_Cl (3 mmol L^–1^), citric acid (31 μmol L^–1^), l-ascorbic acid (8 μmol L^–1^), albumin (3.94 g L^–1^), lysozyme (1.7 g L^–1^) and the pH value was adjusted to 7.4 using Tris HCl (150 mmol L^–1^) and Tris base (150 mmol L^–1^).^32^

### Preparation of fluorescent sensors

A stock solution containing fluorescent diaza-15-crown-5 (MW: 1667.57 g mol^–1^), fluorescent diaza-18-crown-6 (MW: 950.99 g mol^–1^), fluorescent *o*-acetanisidide (MW: 599.67 g mol^–1^), and seminaphtorhodafluor (MW: 453.45 g mol^–1^) were prepared to obtain concentration values ranging from 5.0 to 100.0 μmol L^–1^ in DMSO (organic solvent to prevent hydrolysis of the fluorescent probes). The stock solutions were kept in dark and dry conditions at –20 °C.

### Readouts of fluorescence measurements

A microplate reader was used to measure the fluorescence intensities of the probes in aqueous solution and on G1 paper (Fig. 3–5). Metal ion solutions (25 to 200 mmol L^–1^) in Tris buffer (pH 4.5–9.0, 150 mmol L^–1^) were mixed (1 : 1, v/v) with fluorescent diaza-15-crown-5 (*λ*_ex_/*λ*_em_: 485/528 nm), diaza-18-crown-6 (*λ*_ex_/*λ*_em_: 360/460 nm), *o*-acetanisidide (*λ*_ex_/*λ*_em_: 485/528 nm), and seminaphtorhodafluor (*λ*_ex_/*λ*_em_: 530/590 nm) in DMSO (5.0 to 100.0 μmol L^–1^) and dispensed in 96-well plates (black wall). The excitation and emission peaks were standard microplate reader settings. The calibration of the assay was carried out with Tris buffer (pH 7.4, 150 mmol L^–1^) mixed (1 : 1, v/v) with the probes in DMSO (5.0 to 100.0 μmol L^–1^). The laser-cut round G1 paper (*Ø* = 6.5 mm) was placed at the bottom of the microwells in 96-well plates. Subsequently, fluorescent probe (2 μL) was dropped onto the G1 paper matrix and dried in the air at 24 °C, followed by adding electrolyte solution (2 μL).

### Fabrication of the sample collection device

The sample collection device contained a capillary tube (5 μL) inserted to an Eppendorf tube (0.5 mL) using PDMS sealing. This part was integrated with a paper-based microfluidic device at the other end of the Eppendorf tube.

### Sample preparation of paper-based microfluidic devices

Each fluorescent sensor (diaza-15-crown-5, diaza-18-crown-6, *o*-acetanisidide and seminaphtorhodafluor) (2 μL) was added to the tip of each branch of the paper-based microfluidic device and dried at 24 °C. The paper-based microfluidic device was connected to the sampling device, which was utilized to collect artificial tear fluid by capillary force and dilute the sample. The diluted artificial tear fluid diffused through the main channel of the paper-based microfluidic device to four branches to interact with fluorescent probes in the sensing regions. Additionally, the paper-based microfluidic devices were stored in dry and dark conditions at –20 °C.

### Readout system and image analysis

The paper-based microfluidic device with samples was placed in a portable readout device for imaging. The readout system consisted of eight PMMA pads (5 × 5 × 5 cm^3^). The top part was ablated using a laser beam to form a groove, where a smartphone camera could be placed. The interlayer consisted of a bottom section to fix four LEDs (*λ*_em_ = 508, 366, 460, and 515 nm), a middle glass layer to separate sample and LEDs, and a top layer to fix the sample. A plano-convex lens (*f*: 5.0 cm) was used to change the focus on the smartphone camera. A 3.6 V battery powered the LEDs. Fluorescence images were captured using a smartphone app (exposure time: Shoot 1/4000 s, ISO 400) and analyzed by ImageJ.^33^

### Dilution test

Different volumes of DI water (5, 15, 35, 75, 155, and 315 μL) were added to the reservoir of the sample collection device. Artificial tear fluid (5 μL) was aspirated into the reservoir by using the capillary tube of the sample collection device. The electrolyte sensing measurements were carried out using a custom-made readout device. The images of the fluorescent assays were captured using a smartphone app and processed using ImageJ.

### Evaluation of sample evaporation

G1 paper was cut into 2 mm-wide strips with different lengths (4, 8, 16, and 32 mm). The fluorescent probe (50 μmol L^–1^, 2 μL) was dropped onto the sensing region of the strip and dried at 24 °C for 2 min. Electrolyte solutions (Na^+^ ions (100 mmol L^–1^), K^+^ ions (50 mmol L^–1^), Ca^2+^ ions (1 mmol L^–1^), and Tris buffer (pH = 7.4)) were wicked through the main channel toward the sensing regions of the strips (32 μL).

### Batch-to-batch variation experiments

The concentrations of electrolytes in artificial tear fluid were varied: Na^+^ ions (100–200 mmol L^–1^), K^+^ ions (20–60 mmol L^–1^), Ca^2+^ (0.7–1.0 mmol L^–1^), and pH (7.0–9.0). The quantitative data were obtained using three individual paper-based microfluidic devices.

## Results and discussion

Laser ablation was chosen to pattern the microfluidic channels in paper due to its high speed and accuracy. Filter paper (Whatman grade 1, G1) was patterned by a CO_2_ laser (10.64 μm, 30 W) having a beam spot size of ∼100 μm. This grade is a widely used filter type for routine chemistry applications. As compared to the other filter paper types such as grade 41 (G41), G1 shows rapid filtration time (water flow rate: 57 ml min^–1^) and low autofluorescence (∼30%) (Fig. S1‡). The radiant fluence and beam speed of the CO_2_ laser were optimized at 240 mJ mm^–2^ and 30 mm s^–1^, respectively (Fig. S2‡).

**Fig. 2:**
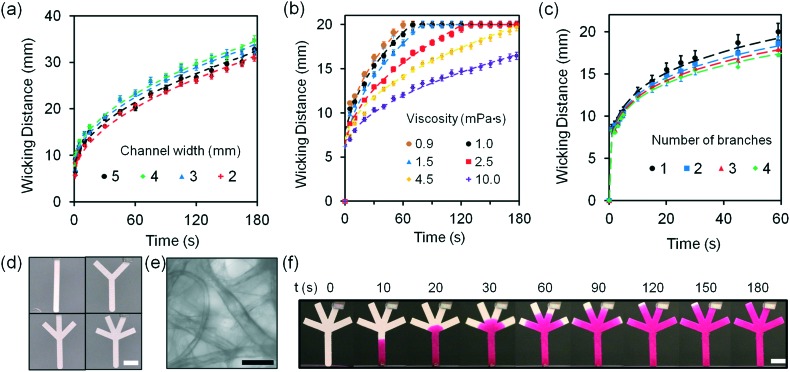
Fluid dynamics characterization of G1 paper-based microfluidic devices at 24 °C. (a) Wicking distances of G1 strips as the width was varied from 2.0 to 5.0 mm. (b) The effect of increase in fluid viscosity from 1.0 to 10.0 mPa s on wicking distance in G1 strips (20 mm in length and 2 mm in width). Fluid viscosities were varied by changing the concentration of glucose (10 μL) from 0.5 to 3.0 mol L^–1^. (c) Wicking distances as the number of branches was increased from 1 to 4 within 60 s. Sample volume was 10 μL. (d) Photographs of paper-based microfluidic devices with different numbers of branches (1 to 4). Scale bar = 4 mm. (e) Bright-field microscopic image of G1 matrix. Scale bar = 50 μm. (f) Photographs of a four-channel paper-based microfluidic device, where Rhodamine B solution (10 mmol L^–1^) was used to show fluid diffusion as a function of time. Scale bar = 4 mm. Error bars represent standard error of the mean (*n* = 3).

To design a paper-based microfluidic device that can operate with tear fluid samples (<10 μL) within a 3 min wicking time (point-of-care application), the geometry of the paper-based microfluidic device was optimized. Upon introducing DI water from their inlets at a constant width (2 mm), the fluid front in G1 strips reached 2 cm within 25 s (Fig. S3‡). As DI water (20 μL) was introduced to G1 strips with different widths at a constant length (3.5 cm), the wicking distances of strips with widths from 2–5 mm were comparable (Fig. 2a). Fig. S4‡ illustrates photographs of the G1 strips with varying channel widths. The G1 strips with 2 mm in width were chosen for assay optimization as it operated at low volume and it was easy to handle. The flow characteristics of fluid were fit using a modified Navier–Stokes equation (eqn (1)).1
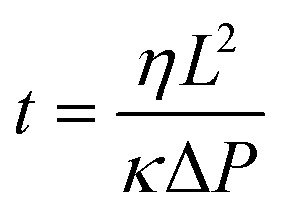
where *t* is the time for the sample to flow to a certain distance, *η* is the viscosity of fluid, *L* is the wicking distance, *κ* is the permeability of fluid, and Δ*P* is applied pressure difference.^34^

The viscosity of human tear fluid ranges from 1 to 10 mPa s.^35^ The G1 matrix should have fast wicking time for application in rapid diagnostics. The viscosities of the solutions (10 μL) introduced to the G1 strips were varied from 1.0 to 10.0 mPa s. While a wicking time of 1 min saturated the fluid front at 1.0 mPa s, fluids with 10.0 mPa s required 3 min for fluid front saturation (Fig. 2b). The optimized G1 strip had fast sample wicking times at viscosities as high as 10.0 mPa s.

Fig. S5‡ illustrates the photographs of G1 microfluidic strips wicking fluids with different viscosities (1.0–10.0 mPa s). The effect of having branched channels on the movement of the fluid front was also tested. As the number of branches was increased from 1 to 4 at a constant main strip width of 2 mm, wicking distances among these strips were comparable within 20 s while and decreased by 14% with increasing number of branches within 20 to 60 s (Fig. 2c). Fig. 2d shows the fabricated paper-based microfluidic devices with different number of branches. The G1 matrix consists of cellulose with porous structure having a particle retention value of 11 μm (Fig. 2e). Fig. 2f shows the photographs of the paper-based microfluidic devices as the Rhodamine B solution (10 mmol L^–1^) was wicked from the main channel. The optimized paper-based microfluidic device with four branches allowed the transport of fluid samples (<20 μL) from the inlet to the branches under 1.5 min (Fig. S6‡).

**Fig. 3:**
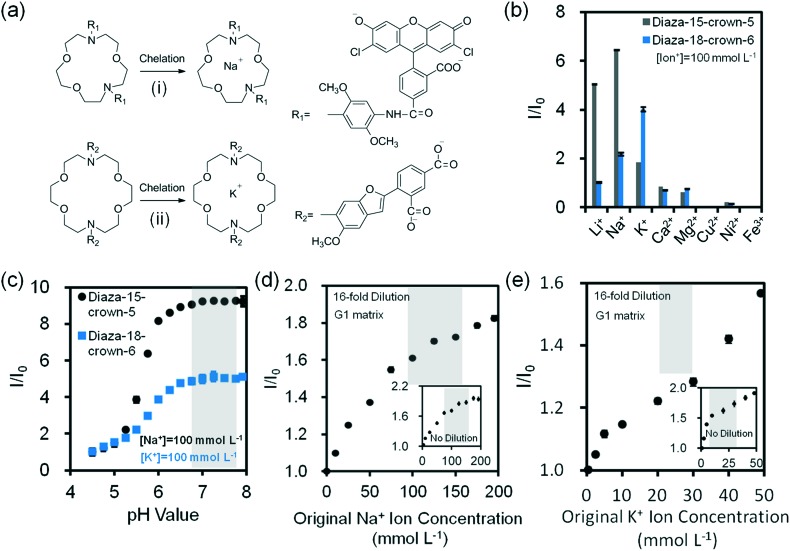
Na^+^ and K^+^ ion measurements using fluorescent crown ether derivatives in buffer solutions (Tris, pH 7.4, 150 mmol L^–1^) at 24 °C. (a) Chelation mechanisms of fluorescent (i) diaza-15-crown-5 and (ii) diaza-18-crown-6 with monovalent metal ions. (b) Selectivities of diaza-15-crown-5 (*λ*_ex_/*λ*_em_: 485/528 nm) and diaza-18-crown-6 (*λ*_ex_/*λ*_em_: 360/460 nm) (25 μmol L^–1^) for mono/divalent ions (100 mmol L^–1^) in aqueous solutions (*n* = 3). (c) The effect of pH on fluorescence readouts at constant Na^+^ and K^+^ ions (100 mmol L^–1^), and diaza-15-crown-5 and diaza-18-crown-6 (25 μmol L^–1^) concentrations in aqueous solutions (*n* = 3). Quantifications of 16-fold diluted (d) Na^+^ and (e) K^+^ ions on G1 matrix at a constant probe concentration (25 μmol L^–1^) (*n* = 6). Insets show the quantifications of non-diluted Na^+^ and K^+^ ions on G1 matrix (*n* = 3). Shadows in Fig. 3c–e show the physiological pH, Na^+^ and K^+^ ion concentration range. Error bars represent standard error of the mean.

Assay conditions of fluorescent chelating agents were optimized for functionalizing paper-based microfluidic devices. Crown ethers are cyclic chelating agents that are specific to monovalent metal ions.^36^ They form stable complexes with cations by ion–dipole interaction between a metal ion and negatively charged oxygen atoms in the polyether ring.^37^ The changes in fluorescence intensity on monovalent metal ion binding are caused by conformational or electronic changes that possibly occur in electron transfer between ground state and excited state of fluorophore due to electron density changes at the ion binding site.^38,39^ Fluorescent diaza-15-crown-5 (cavity size: 0.17–0.22 nm) and diaza-18-crown-6 (cavity size: 0.26–0.32 nm) were utilized for selectively sensing Na^+^ and K^+^ ions (Fig. 3a). In the presence of diaza-15-crown-5 (25 μmol L^–1^) in buffer solution (Tris, pH 7.4, 150 mmol L^–1^) at 24 °C, Na^+^ ions (100 mmol L^–1^) had the highest fluorescence intensity due to the cavity specific 1 : 1 chelation (Fig. 3b). The diaza-15-crown-5 response to Na^+^ ions was 3.5 fold higher as compared to K^+^ ions. In the presence of diaza-18-crown-6, K^+^ ions showed the highest selectivity (Fig. 3b). The diaza-18-crown-6 selectivity to K^+^ ions was 1.8 fold higher than Na^+^ ions. The interference due to Na^+^ ions could be due to 2 : 1 complexation with diaza-18-crown-6 cavity. To analyze ion binding affinity, the dissociation constants (*K*_d_, the ion concentration at which 50% of crown ethers (receptors) are chelated by ions) of Na^+^ and K^+^ binding to fluorescent crown ether derivatives were determined as 14 mmol L^–1^ and 5.8 mmol L^–1^ respectively in Tris buffer solution (150 mmol L^–1^, pH = 7.4):2
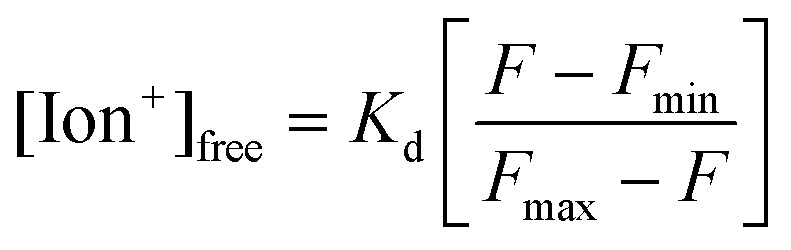
where [Ion^+^]_free_ is the free ion concentration of the solution, *F* is the fluorescence intensity at a given ion concentration, *F*_min_ is the fluorescence intensity at the ion-free concentration, and *F*_max_ is the fluorescence intensity at the ion-saturated concentration. *K*_d_ for Na^+^ and K^+^ ions match the previously reported values for Na^+^ ions (5–20 mmol L^–1^) and K^+^ ions (5.1 mmol L^–1^) of in the physiological range (pH 6–8).^38^ The decrease in the emission intensity of aqueous diaza-15-crown-5 and diaza-18-crown-6 probes in the presence of Cu^2+^, Ni^2+^, and Fe^3+^ ions can be attributed to orbital energy gaps of these ions that absorb the excitation light and consequently reduce the emission intensity.

Increasing pH from 7.0 to 8.0 enhanced the fluorescence intensities 0.2% and 2.6% for diaza-15-crown-5 and diaza-18-crown-6 respectively, showing stability within the physiological pH range (∼7.4) of tear fluid (Fig. 3c). The fluorescence intensities of both diaza-15-crown-5 and diaza-18-crown-6 depended on their concentrations. As their concentrations increased from 3 μmol L^–1^ to 50 μmol L^–1^ in buffer solutions (Tris-buffer, pH 7.4, 150 mmol L^–1^), their fluorescence intensities increased 10 and 13 fold, respectively (Fig. S7‡). When the temperature increased from 25 to 40 °C, the fluorescence intensity of diaza-15-crown-5 decreased ∼20% (Fig. S8‡). However, the diaza-18-crown-6 fluorescence intensity variation was ∼2% between 25 to 35 °C, decreasing ∼9% at 40 °C (Fig. S8‡).

The concentration of Na^+^ ions in tear fluid of healthy individuals is 120–165 mmol L^–1^.^30^ In dry eye syndrome caused by MGD and LGD, tear Na^+^ ion concentration increases by 2.2% and 6.8%, respectively.^31^ This requires a sensor sensitivity of ∼3.0 mmol L^–1^ and ∼9.0 mmol L^–1^. The presence of both MGD and LGD increases the Na^+^ ion concentration by 8.9%, requiring a sensitivity of ∼12.0 mmol L^–1^.^40^ G1 paper (32 mm^2^) was used as a reaction matrix to quantify concentrations of electrolytes. Probe solutions (2 μL, 25 μmol L^–1^ in DMSO) were immobilized on the G1 matrix, followed by adding ion solutions (2 μL, Tris-buffer, pH 7.4, 150 mmol L^–1^) to the G1 matrix. The fluorescent probes dissolved DMSO (2 μL) were inoculated on G1 paper, which was dried at 24 °C for 2 min (Fig. S9‡). As the concentration of Na^+^ ions in the presence of diaza-15-crown-5 on G1 matrix increased from ion-free buffer solution (Tris, pH 7.4, 150 mmol L^–1^) to 200 mmol L^–1^, the fluorescence intensity of the probe increased by 1.9 fold (Fig. 3d inset). To detect Na^+^ ions within the physiological concentration range of tear fluid (100–200 mmol L^–1^), the samples were diluted 16 fold (4 serial two-fold dilutions). After dilution, the fluorescence intensity of probes within the physiological Na^+^ ion range (100–200 mmol L^–1^) increased 13.3% (Fig. 3d and S10a‡). The sensitivity of the diluted Na^+^ ion sensor on G1 matrix was calculated to be 1.5 mmol L^–1^, which met the requirement for the diagnosis of dry eye. Sensitivity values from three/six independent measurements were calculated by averaging the standard error of the intensity ratio (*I*/*I*_0_) on the slope within the physiological disease detection range, followed by reading the corresponding electrolyte concentration values (mmol L^–1^) in the *x*-axis (ESI‡ Fig. S11).

The concentration of K^+^ ions in tear fluid of healthy individuals is 20–42 mmol L^–1^.^30^ However, in dry eye syndrome caused by MGD and LGD, tear K^+^ ion concentration increases by 2.5% and 3.8%, respectively.^40^ This requires a sensor sensitivity of ∼0.6 mmol L^–1^ and ∼0.9 mmol L^–1^. The presence of both MGD and LGD increases the K^+^ ion concentration by 5.8%, requiring a sensitivity of ∼1.4 mmol L^–1^. As the concentration of K^+^ ions increased from ion-free to 100 mmol L^–1^ at 24 °C, fluorescence intensity of diaza-18-crown-6 (25 μmol L^–1^) increased 97.7% (Fig. 3e inset). To detect K^+^ ions within the physiological range of tear fluid (20–50 mmol L^–1^), the samples were diluted 16 fold. After dilution, the fluorescence intensity of the probe within the physiological range increased 28.3% (Fig. 3e and S10b‡). The sensitivity of K^+^ ion sensor was 0.9 mmol L^–1^ which met the requirement for the diagnosis of dry eye.

**Fig. 4:**
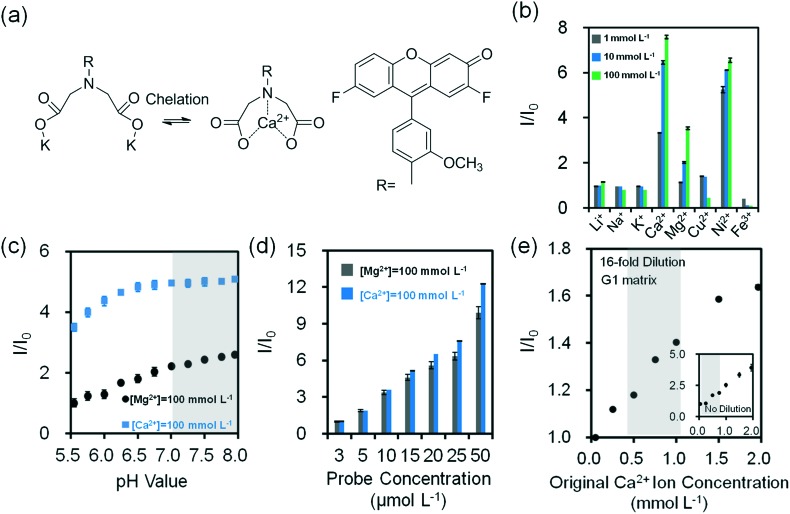
Quantification of divalent metal ions in buffer solution (Tris, pH 7.4, 150 mmol L^–1^) at 24 °C. (a) Chelation mechanism of *o*-acetanisidide with divalent metal ions. (b) Fluorescence readouts of *o*-acetanisidide (*λ*_ex_/*λ*_em_: 485/528 nm) in the presence mono/divalent metal ions in aqueous solutions (*n* = 3). (c) The effect of pH on fluorescence readouts of *o*-acetanisidide (25 μmol L^–1^) at constant Mg^2+^ and Ca^2+^ ion concentrations (100 mmol L^–1^) in aqueous solutions (*n* = 3). (d) Fluorescence intensity readouts of Mg^2+^ and Ca^2+^ ions (100 mmol L^–1^) as the concentration of *o*-acetanisidide were varied from 3–50 μmol L^–1^ (*n* = 3). (e) Quantification of 16-fold diluted Ca^2+^ ions on G1 matrix at a constant *o*-acetanisidide concentration (25 μmol L^–1^) (*n* = 6). Insets show the quantification of non-diluted Ca^2+^ ions on G1 matrix (*n* = 3). Shadows in Fig. 4c and e show the physiological pH and Ca^2+^ ion concentration range. Error bars represent standard error of the mean.

To sense divalent metal ions, *o*-acetanisidide was utilized, where the *N*-(2-methoxyphenyl)iminodiacetate served as a generic chelation site (Fig. 4a). Among mono/divalent ions (1–100 mmol L^–1^) in the presence of *o*-acetanisidide (25 μmol L^–1^, Tris buffered, pH 7.4, 150 mmol L^–1^) at 24 °C, Ca^2+^ ions (100 mmol L^–1^) had the highest fluorescence intensity (1.2 fold higher than Ni^2+^ ions and 2.1 fold higher than Mg^2+^ ions), due to the site specific 1 : 1 chelation (Fig. 4b). The dissociation constant of Ca^2+^ ions was calculated to be 0.9 mmol L^–1^ (eqn (2)). The chelation of divalent metal ions depended on the pH; increasing the pH value from 5.5 to 8.0 enhanced the fluorescence intensity 2.6 and 1.5 fold for *o*-acetanisidide in the presence of Mg^2+^ and Ca^2+^ ions, respectively, showing stability within the physiological pH range (∼7.4) of tear fluid (Fig. 4c). As the concentration of *o*-acetanisidide was increased from 3 μmol L^–1^ to 50 μmol L^–1^, the fluorescence intensities for Mg^2+^ and Ca^2+^ ions increased 9.9 and 12.3 fold, respectively (Fig. 4d). The fluorescence intensity was affected by temperature; for example, when temperature increased from 25 °C to 40 °C, the fluorescence intensity of *o*-acetanisidide decreased ∼25% (Fig. S12a‡).

The concentration of Ca^2+^ ions in tear fluid of healthy individuals is 0.4–1.1 mmol L^–1^.^42^ However, in dry eye syndrome caused by MGD or LGD, tear Ca^2+^ ion concentration increases 2.5% and 5.0%, respectively. This requires a sensor sensitivity of 0.02–0.04 mmol L^–1^. The presence of both MGD or LGD increases the Ca^2+^ ion concentration 7.5%, requiring a sensitivity of ∼0.06 mmol L^–1^.^40^ As the concentration of Ca^2+^ ions increased from 0.25 mmol L^–1^ to 1.5 mmol L^–1^ at 24 °C, fluorescence intensity increased 3 fold in the presence of *o*-acetanisidide (25 μmol L^–1^) (Fig. 4e inset). The high sensitivity range of the fluorescent *o*-acetanisidide is 0.25–1.5 mmol L^–1^. To detect Ca^2+^ ions within the physiological concentration range of tear fluid, the sample does not need to be diluted. Even after 16-fold dilution, the fluorescence intensity of Ca^2+^ ion solution (0.25 mmol L^–1^ to 1.50 mmol L^–1^) increased 47% on the G1 matrix (Fig. 4e and S10c‡). The sensitivity of the Ca^2+^ ion sensor was calculated to be 0.03 mmol L^–1^ which met the requirements of dry eye diagnostic sensitivity.

**Fig. 5:**
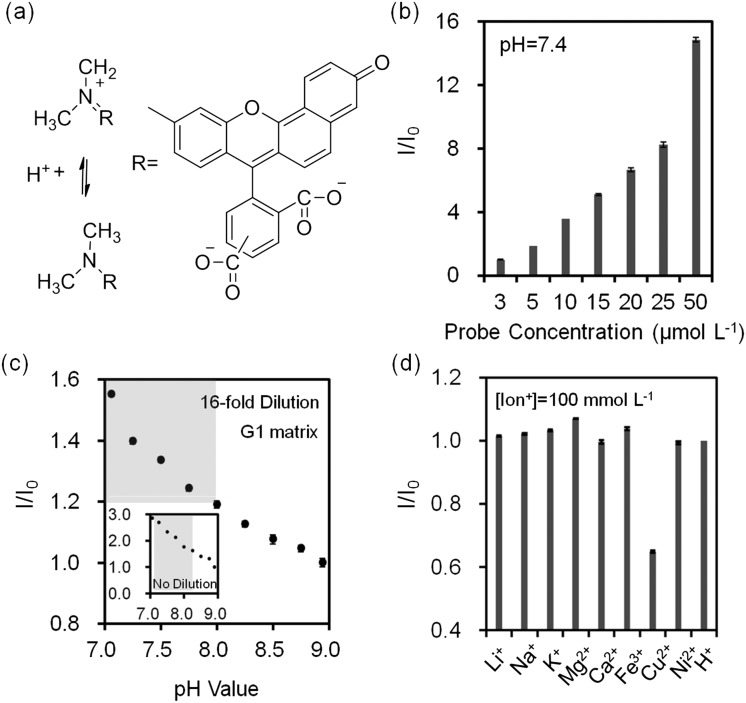
Quantification of pH values in buffer solutions (Tris, 150 mmol L^–1^) at 24 °C. (a) Principle of operation of seminaphtorhodafluor. (b) Fluorescence intensity readouts as the concentration of seminaphtorhodafluor (*λ*_ex_/*λ*_em_: 530/590 nm) was varied from 3–50 μmol L^–1^ at pH = 7.4 in aqueous solutions (*n* = 3). (c) Quantification of Tris buffer pH (inset) and 16-fold diluted Tris buffer (150 mmol L^–1^, pH = 7.4) on G1 matrix at a constant seminaphtorhodafluor concentration (25 μmol L^–1^) (*n* = 6). Insets show the quantification of non-diluted Tris buffer on G1 matrix (*n* = 3). Shadows in Fig. 5c show the physiological pH range. (d) Relative fluorescence intensity readouts of mono/divalent ions at a constant concentration of seminaphtorhodafluor (25 μmol L^–1^) at pH 7.4 (*n* = 3). Error bars represent standard error of the mean.

pH changes can be quantified using seminaphtorhodafluor (p*K*_a_ value ∼7.5), the fluorescence emission shifts from yellow-orange (*λ* = 580 nm) to deep red (*λ* = 640 nm) under acidic and basic conditions, respectively (Fig. 5a). As the concentration of seminaphtorhodafluor was increased from 3 μmol L^–1^ to 50 μmol L^–1^ at a constant pH value (7.4), the fluorescence intensity increased 15 fold (Fig. 5b). The tear fluid pH of a healthy individual is ∼7.4; however, in dry eye (MGD and LGD) the pH increases to ∼7.9.^41^ Changes in the composition and/or concentration of mucin secreted by the goblet cells increase the pH of the overlying aqueous layer.^41^ This requires a sensor sensitivity of ∼0.5 pH units. The seminaphtorhodafluor on G1 matrix exhibited a fluorescence intensity decrease of 2.9 fold as the pH increased from 7.0 to 9.0 (Fig. 5c inset). The sensitivity of seminaphtorhodafluor was 0.06 pH units after a 16-fold dilution, which met the requirement of the sensor sensitivity (Fig. 5c and S10d‡). Seminaphtorhodafluor also showed low interference in the presence of mono/divalent ions in solution (Fig. 5d). Additionally, the fluorescence intensity of seminaphtorhodafluor decreased ∼36% when the temperature increased from 25 °C to 40 °C (Fig. S12b‡).

To investigate potential ion interference in the quantification of electrolyte concentrations, the 16-fold diluted solutions containing two or more ions both in solution and on the G1 matrix were analyzed (Fig. S13‡). Fluorescence intensity changes of diaza-15-crown-5 for Na^+^ ion (30–180 mmol L^–1^) sensing in the presence of K^+^ (42 mmol L^–1^), Ca^2+^ (1.1 mmol L^–1^), and Mg^2+^ (0.4 mmol L^–1^) ions were evaluated. The maximum deviation for the interference of K^+^ ions in Na^+^ ion sensing was 2.4% in solution and 4.0% on the G1 matrix. For Na^+^ ion sensing interfered by K^+^ and Ca^2+^ ions, the deviation was 5.0% in solution and 2.8% on the G1 matrix. Additionally, in the presence of K^+^, Ca^2+^ and Mg^2+^ ions, Na^+^ ion measurement interference was 2.7% in solution and 4.7% on the G1 matrix. In the physiological range of human tear fluid (120–180 mmol L^–1^), the deviations of Na^+^ ion measurements were less than 1.4%. The deviations for Na^+^ ion sensing from the results of fluorescent crown ether derivatives were within the accuracy limit of target selectivities. Moreover, ion sensing in artificial tear fluid was evaluated and compared with electrolyte solutions in buffers (Fig. S14‡). Artificial tear fluid was prepared to mimic tear fluid composition. The maximum deviations for Na^+^ ions (0–200 mmol L^–1^), K^+^ ions (0–50 mmol L^–1^), Ca^2+^ ions (0–2 mmol L^–1^), pH (7.0–9.0) sensing on the G1 matrix were 5%, 5%, 6% and 5%, respectively, which were within the accuracy of target electrolyte sensing. Therefore, these fluorescent sensors can be used for ion sensing in artificial tear fluid on the G1 matrix.

**Fig. 6:**
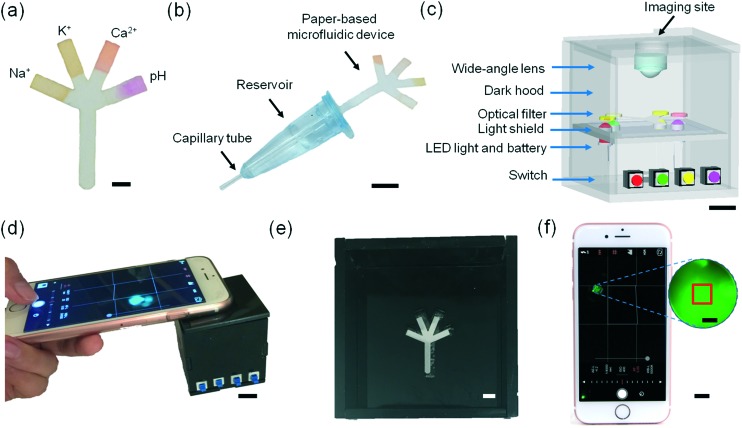
Microfluidic system for tear fluid analysis. (a) Paper-based microfluidic device impregnated with fluorescent probes. Scale bar = 2 mm. (b) Sample collection and dilution device using a capillary tube. Scale bar = 1 cm. (c) The schematic of the portable readout device. Scale bar = 1 cm. (d) The use of the portable readout device for capturing the image of the fluorescent probes. Scale bar = 1 cm. (e) Photograph of the interlayer groove to place the paper-based microfluidic device. Scale bar = 4 mm. (f) Screenshot of the smartphone app capturing an assay image. Scale bar = 1 cm. Red square (1 × 1 mm^2^) in the magnified screenshot (blue dashes) shows the selected sensing region. Scale bar = 1 mm.

To demonstrate the utility of the paper-based microfluidics for tear analysis, a microfluidic system including a sample collection device and a portable readout device was developed. 2 μL of each fluorescent sensor (Na^+^, K^+^, Ca^2+^ ions and pH sensors) was dispensed onto the tip of each branch of the microfluidic device (Fig. 6a and movie S1‡). The sample collection device was designed to be amenable to potential clinical use consisting of a dilution reservoir (∼75 μL DI water) connected to a capillary tube, which can sample tears (∼5 μL) (Fig. S15‡). The diluted sample was mixed thoroughly (30 s) and introduced to the paper-based microfluidic device which was connected to the opposite side of the reservoir (Fig. 6b and movie S2‡). A portable readout device was developed for blocking the ambient light and exciting the fluorescent probes impregnated into the branches of the paper-based microfluidic device (Fig. 6c). Four LEDs with different emission wavelengths (*λ*_em_: 366, 460, 505, and 515 nm) illuminated the sensing regions from the rear. The four-channel paper-based microfluidic device was placed in a groove covered with a longpass filter, which was located in the interlayer of the readout device. Fig. S16‡ shows light attenuation of each LED light using the longpass filters (420, 495, 515, and 590 nm). The fluorescence images of probes at different artificial tear fluid compositions on paper-based microfluidic device were captured by an iPhone 6S camera positioned over a wide-angle lens in the readout device using a smartphone app (Shoot) (Fig. 6d–f and S17‡). A square (1 × 1 mm^2^) in the central of the captured fluorescence image was selected from the sensing region (2 × 2 mm^2^) at the end of each branch of the paper-based microfluidic device, which was used for image processing (Fig. 6f inset). Movie S3‡ shows the operation of the readout device for sample measurements.

The quantification of electrolyte concentrations in the artificial tear fluid was carried out by a portable readout device integrated with a smartphone camera. The fluorescence images were captured using a smartphone app and quantitatively analyzed using ImageJ. A square (1 × 1 mm^2^) in the central of the captured fluorescence image was selected from the sensing regions (2 × 2 mm^2^) at the end of each branch of the paper device for signal processing. The concentration-dependent fluorescence intensity ratio can be expressed as:3
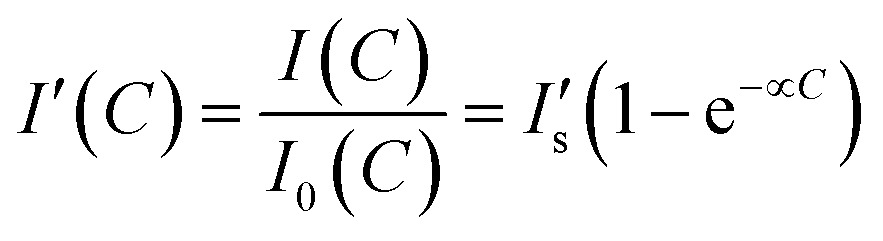
where 
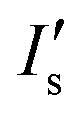
 represents the saturated fluorescent intensity ratio, ∝ is the saturation decay constant, and *C* is the electrolyte concentration. Eqn (3) was used to express fluorescence intensity as a function of electrolyte concentration.

**Fig. 7:**
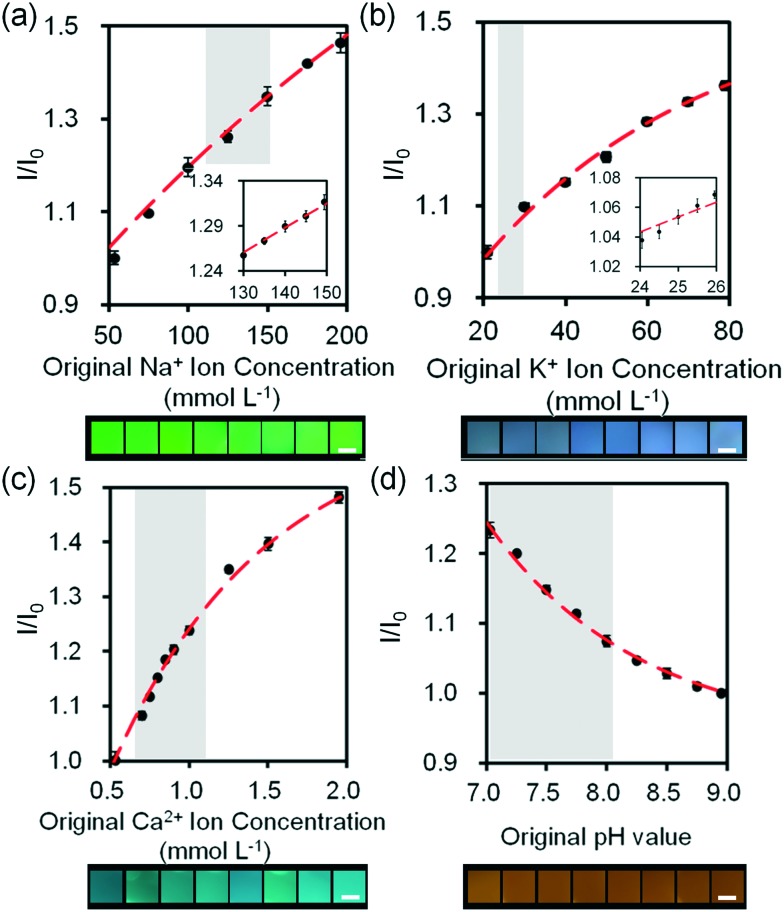
Quantifications of electrolytes in artificial tear fluid using the smartphone readout system: (a) Na^+^ ions, (b) K^+^ ions, (c) Ca^2+^ ions and (d) H^+^ ions sensing. Scale bars = 2 mm. Insets in (a) and (b) show Na^+^ ion concentration in the range of 130–150 mmol L^–1^ and K^+^ ion concentration in the range of 24–26 mmol L^–1^. Error bars represent standard error of the mean (*n* = 6). Curves (red dashes) were fitted using eqn (3). Shadows show the physiological Na^+^, K^+^, Ca^2+^ ion concentration and pH ranges.

We investigated the sensitivity of electrolyte sensing based on calibration curves (Fig. 7 and S18‡). The calibration data was compiled by subtracting the background (paper without fluorescent probe) (eqn S1‡). Increase in Na^+^ ion concentration from 100 mmol L^–1^ to 200 mmol L^–1^ in artificial tear fluid within the physiological range increased the fluorescence intensity of diaza-15-crown-5 by 22.4% on the paper-based microfluidic device (Fig. 7a). The sensitivity of diaza-15-crown-5 sensor on the microfluidic device was 2.7 mmol L^–1^, which met the requirement for Na^+^ ion sensing in dry eye diagnosis (∼3.0 mmol L^–1^). Fig. S19‡ shows the reproducibility of the sample measurement process using the sample collection device (Fig. 6b) that performed 2 to 64 fold dilutions. The average measurement error due to sample dilution in Na^+^ ions was 1.3 mmol L^–1^ (Fig. S19a‡). As the concentration of K^+^ ions was increased within the physiological range in artificial tear fluid (20 mmol L^–1^ to 50 mmol L^–1^), the fluorescence intensity of diaza-18-crown-6 sensor on the microfluidic device increased by 26.6% (Fig. 7b). The sensitivity of diaza-18-crown-6 sensor was 1.4 mmol L^–1^, which met the requirement for K^+^ sensing in dry eye diagnosis (∼1.4 mmol L^–1^). The average measurement error due to sample dilution in K^+^ ions was 0.8 mmol L^–1^ (Fig. S19b‡). Additionally, as the concentration of Ca^2+^ ions in artificial tear fluid was increased from 0.5 mmol L^–1^ to 2.0 mmol L^–1^, the fluorescence intensity of *o*-acetanisidide sensor on the microfluidic device increased 80.4% (Fig. 7c). The sensitivity of *o*-acetanisidide was 0.02 mmol L^–1^, which met the requirement for Ca^2+^ sensing (0.02–0.04 mmol L^–1^) in dry eye diagnosis. The average measurement error due to sample dilution in Ca^2+^ ions was 0.02 mmol L^–1^ (Fig. S19c‡). An increase in pH value from 7.0 to 8.0 in artificial tear fluid decreased the fluorescence intensity of seminaphtorhodafluor by 18.9% on the microfluidic device. The average measurement error due to sample dilution was 0.1 pH values (Fig. S19d‡). The sensitivity was calculated to be 0.06 pH units, which met the requirement for pH sensing in dry eye diagnosis (∼0.5 pH unit) (Fig. 7d). The fluorescence intensity measurements of paper-based microfluidic device using the readout system integrated with the smartphone app and ImageJ were consistent with the results from the microplate reader. The paper-based microfluidic devices remained exposed to air during measurements. We performed experiments to measure the effect of evaporation on the fluorescence intensity readouts in paper strips with different lengths (4, 8, 16, and 32 mm) during the wicking process. The evaporation from the electrolyte solutions during wicking process on paper device did not have significant effect on the fluorescence readouts (Fig. S20‡). The average standard errors due to evaporation among different strips were 0.08, 0.07, 0.02 mmol L^–1^, and 0.07 pH units for Na^+^, K^+^, Ca^2+^, and pH measurements, respectively. Additionally, batch-to-batch measurements of electrolytes in the paper-based microsystem showed that the average detection errors were 1.0 mmol L^–1^ (Na^+^ ions), 1.3 mmol L^–1^ (K^+^ ions), 0.02 mmol L^–1^ (Ca^2+^ ions), and 0.13 pH, indicating high reproducibility in independent trials (Fig. S21‡).

**Fig. 8:**
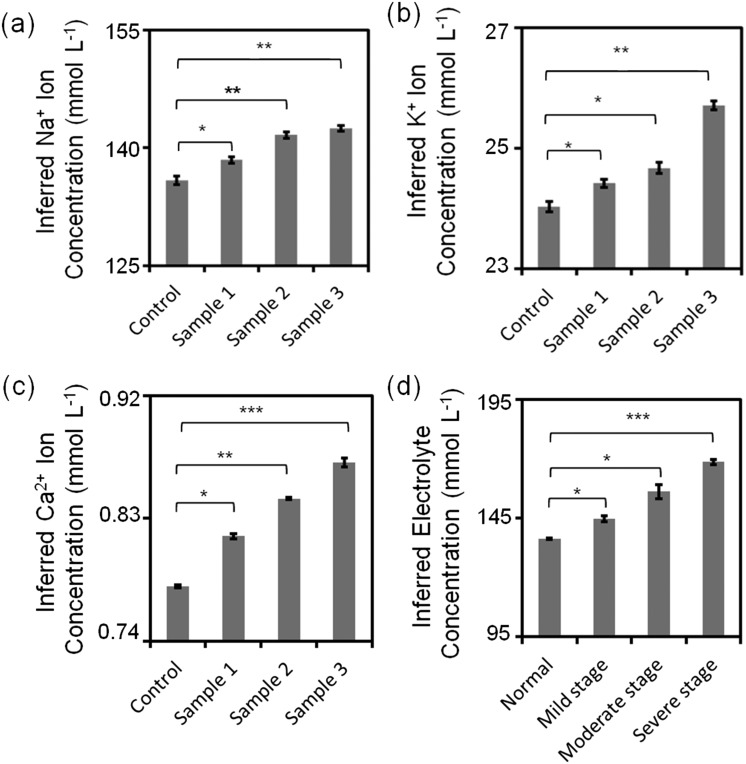
Quantitative analysis of simulated artificial tear samples. (a) Sub-type differentiation of dry eye: inferred (a) Na^+^, (b) K^+^, (c) Ca^2+^ ion concentrations and (d) different stages of dry eye. *: *p* < 0.05, **: *p* < 0.01, ***: *p* < 0.001, compared with control. Error bars represent standard error of the mean (*n* = 3).

Sub-types of dry eye (MGD and LGD) were simulated in artificial tear fluid by varying the concentrations of Na^+^, K^+^, and Ca^2+^ ions (Table 1). Fig. 8a–c shows the variation of inferred ion concentration in artificial tear samples. Fig. S22‡ shows the measurements of pH values. Ion concentrations in artificial tear sample 1–3 (simulated dry eye samples) were higher than that in control (simulated healthy sample). The maximum deviation of Na^+^ ion sensor was calculated to be 3% according to the standard curve, which was within the accuracy for dry eye diagnosis (∼3%). Moreover, the maximum deviation of the fluorescent sensors for K^+^, Ca^2+^ ions and pH were 3%, 0.4%, and 4%, respectively, which were within the accuracy for dye eye diagnosis (∼7%). Additionally, total electrolyte concentration of human tear fluid is correlated with tear osmolarity, which increases with dry eye severity.^42^ Different severity stages of dry eye were simulated by varying the concentrations of Na^+^, K^+^, and Ca^2+^ ions (Table 1) on the paper-based microfluidic device. The ion concentrations and pH value were measured in the portable readout device. The images were captured using a smartphone app and analyzed by ImageJ. The maximum deviation of the sensor in all samples was 1.4%, which was within the accuracy for dry eye diagnosis (∼2%) (Fig. 8d).

**Table 1:** Ion concentrations in artificial tear samples from sub-types and different severity stages

Subtype-differentiation				
Ions	Control (mmol L^–1^)	Sample 1 (MGD) (mmol L^–1^)	Sample 2 (LGD) (mmol L^–1^)	Sample 3 (MGD + LGD) (mmol L^–1^)
Na^+^	133.2	136.1	142.2	145.1
K^+^	24.0	24.6	24.9	25.4
Ca^2+^	0.80	0.82	0.84	0.86

Severity stages				
Ions	Normal (mmol L^–1^)	Mild stage (mmol L^–1^)	Moderate stage (mmol L^–1^)	Severe stage (mmol L^–1^)
Na^+^	135	145	155	165
K^+^	24	25	26	27
Ca^2+^	0.80	0.85	0.90	0.95

## Conclusions

We have developed paper-based microfluidics and system integration strategies for a new class of dry eye diagnostics based on fluorescent chelating agents. The microfluidic system containing a capillary sample collection tube and portable readout device shows high selectivity and sensitivity in detecting tear electrolytes. The paper-based microfluidic sensor and the readout device allow for quantification of the fluorescence signal to report on the concentration of electrolytes using a smartphone for application in the diagnosis of dry eye at point-of-care settings. Sensing electrolytes in simulated human tear fluid using our microfluidic system provides diagnostic information on the sub-types of dry eye and their severity within 3 min. The miniaturized microfluidics and portable readout device highlight the practical applicability and effectiveness of the microfluidic system for dry eye diagnostics. This microfluidic system may provide new opportunities for the diagnosis and differentiation of ocular disease, such as MGD and LGD. The future directions of our present work in translation to clinical settings include testing with patient samples and improving sensor accuracy and reliability.

## Author contributions

A. K. Y. conceived the idea. N. J., A. K. Y., and A. G. performed the experiments. A. K. Y. and N. J. wrote the manuscript. S. H. Y., A. K., S. M. P., G. U. R. E., H. B., Y. Z. S., J. S. W. and A. T. made intellectual contributions and revised the manuscript. H. B. thanks Wellcome Trust (201929/Z/16/Z) and Leverhulme Trust (RF-2016-039) for funding.
