# Supplementary material for: Paper-based microfluidic system for tear electrolyte analysis
Source: Lab Chip. 2017 Feb 16;17(6):1137–48. doi: 10.1039/c6lc01450j (PMC5433427; doi:10.1039/c6lc01450j)
Supplement: Supplementary file 4 [file LC-017-C6LC01450J-s004.pdf]

Electronic Supplementary Material (ESI) for Lab on a Chip.
This journal is © The Royal Society of Chemistry 2017

SUPPORTING INFORMATION

Paper-Based Microfluidic System for Tear Electrolyte Analysis

Ali K. Yetisen,§*[ab] Nan Jiang,§[abc] Ali Tamayol,[ab] Guillermo U. Ruiz-Esparza,[ab] Yu Shrike Zhang,[ab] Sofía Medina-Pando,[a] Aditi Gupta,[b] James S Wolffsohn,[d] Haider Butt,[e] Ali Khademhosseini,[abfg] and Seok-Hyun Yun *[bh]

[a] Biomaterials Innovation Research Center, Division of Biomedical Engineering, Brigham and Women's Hospital, Harvard Medical School, Cambridge, Massachusetts, 02139, USA;

[b] Harvard-MIT Division of Health Sciences and Technology, Massachusetts Institute of Technology, Cambridge, Massachusetts 02139, USA

[c] State Key Laboratory of Advanced Technology for Materials Synthesis and Processing, Wuhan University of Technology, 122, Luoshi Road, Wuhan, 430070, China

[d] Ophthalmic Research Group, School of Life and Health Sciences, Aston University, Aston Triangle, Birmingham B4 7ET, UK

[e] Nanotechnology Laboratory, School of Engineering, University of Birmingham, Birmingham B15 2TT, UK

[f] Department of Physics, King Abdulaziz University, Jeddah, 21589, Saudi Arabia;

[g] Department of Bioindustrial Technologies, College of Animal Bioscience and Technology, Konkuk University, Hwayang-dong, Gwangjin-gu, Seoul 143-701, Republic of Korea

[h] Harvard Medical School and Wellman Center for Photomedicine, Massachusetts General Hospital, 65 Landsdowne Street, Cambridge, Massachusetts 02139, USA

* E-mail: akyetisen@gmail.com, syun@mgh.harvard.edu

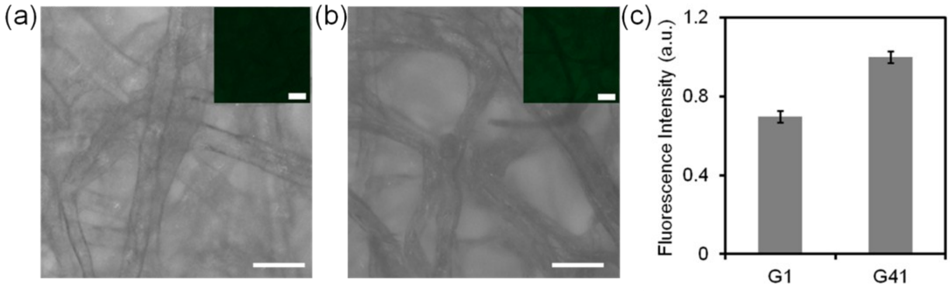

Figure S1. Microscopic images of (a) G1 paper and (b) G41 paper under brightfield. Insets show the fluorescence image of G1 and G41 (λ_{ex}: 488 nm). Scale bar=50 μm. (c) Quantification of fluorescence intensity of G1 and G41. Error bars represent standard error of the mean (n=3).

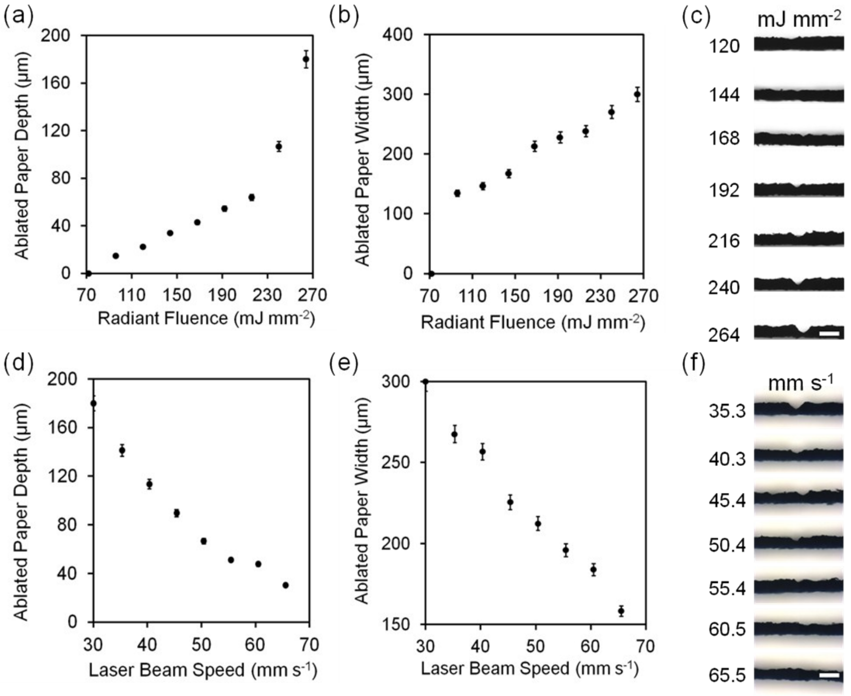

Figure S2. Optimization of CO_2 laser radiation fluence and beam speed for ablating filter paper-G1 (nominal thickness=180 μm). (a) Depth and (b) width at a constant beam speed (40.3 mm s^{-1}). (c) Photographs of laser ablated matrix cross-sections of G1 at a constant beam speed. Scale bar=200 μm. (d) Depth and (e) width of the ablated regions as the laser beam speed was varied at a constant fluence (240 mJ mm-

^{2}). (f) Photographs of laser ablated matrix cross-sections of G1 matrix at a constant fluence (n=3). Scale bar=200 μm. Error bars represent standard error of the mean (n=3).

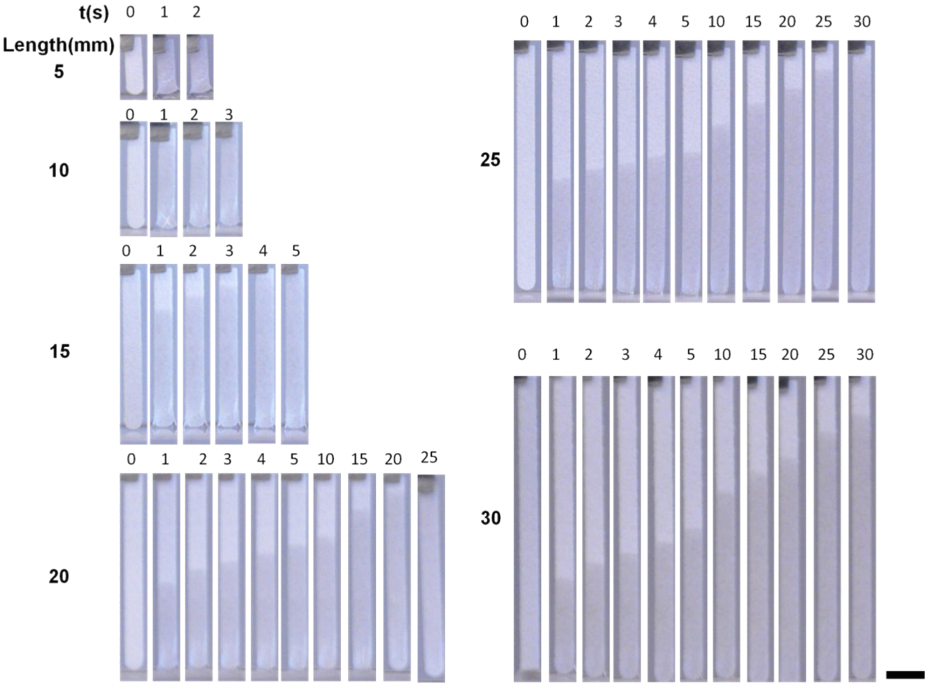

Figure S3. Photographs of DI water diffusion on microfluidic channels with different lengths at a constant width (2 mm) at 24 °C. Scale bar=4 mm.

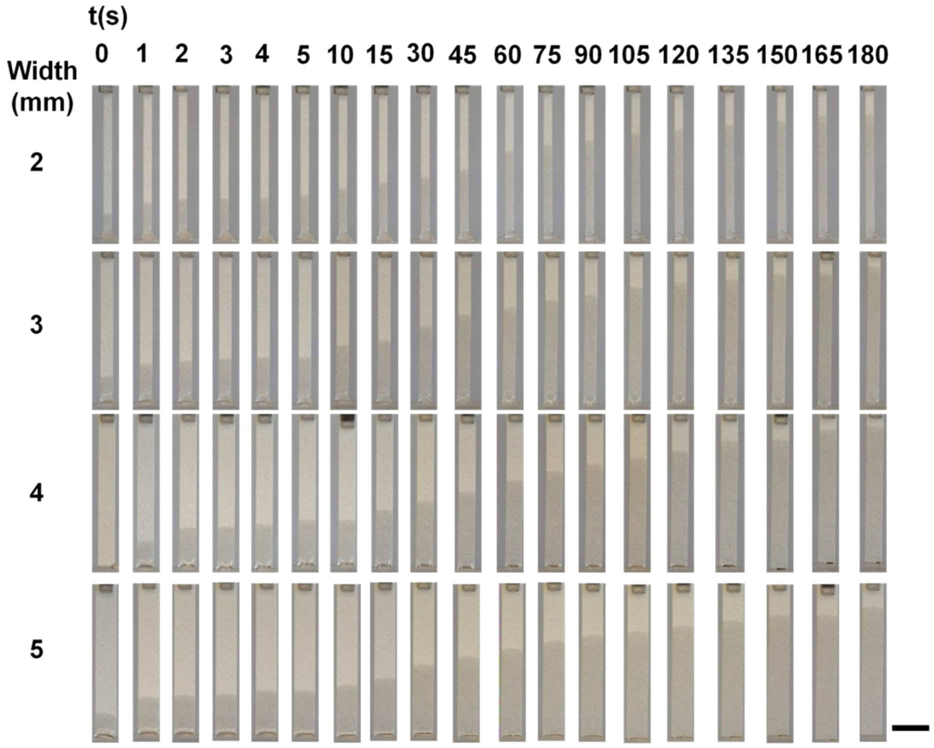

Figure S4. Photographs of DI water (20 μL) diffusion on microfluidic channels with different widths at a constant length (2 mm) at 24 °C. Scale bar=4 mm.

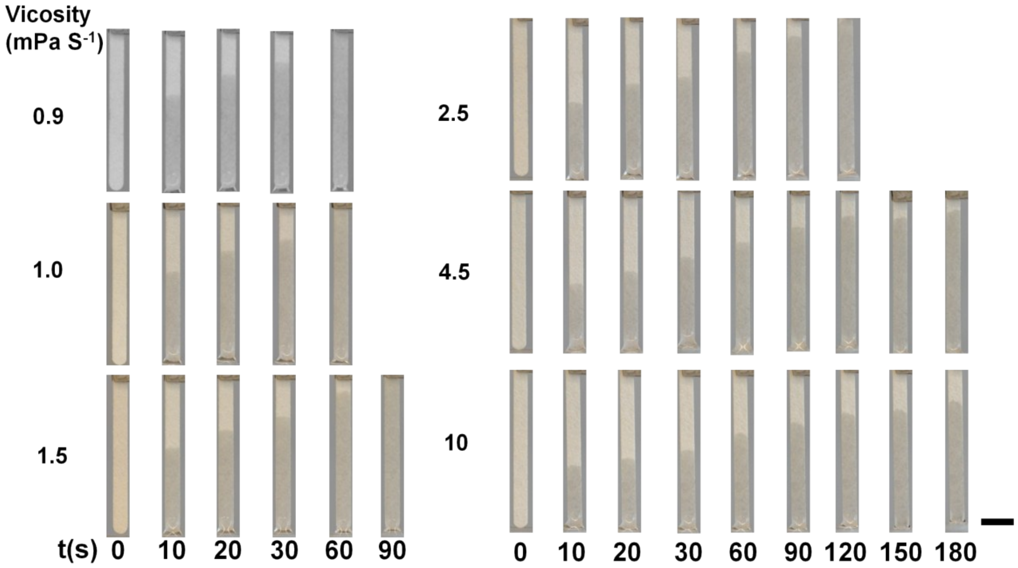

Figure S5. Photographs of increase in fluid viscosity from 1.0 to 10.0 mPa s^{-1} on G1 strip (20 mm × 2 mm). Scale bar=4 mm.

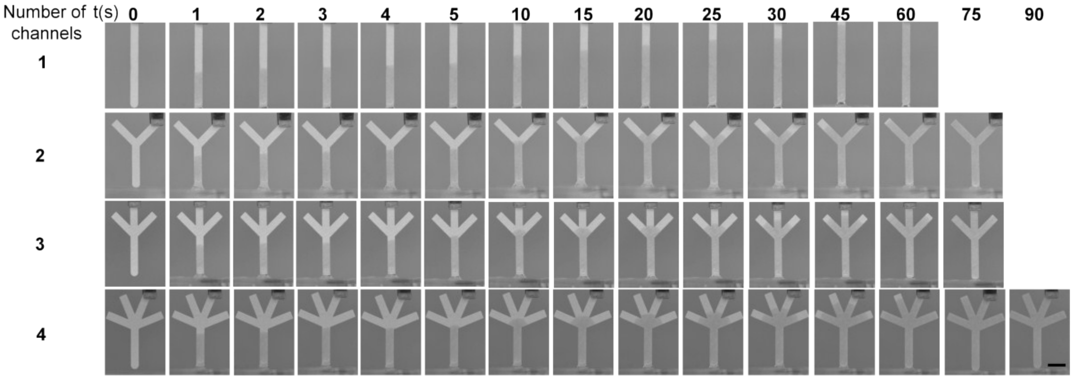

Figure S6. Photographs of increase in channels from 1 to 4 on G1matrix. Scale bar=4 mm.

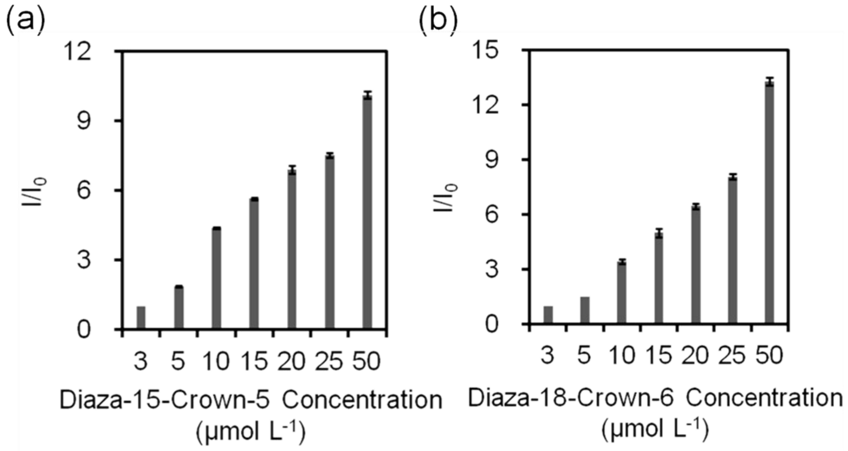

Figure S7. Fluorescence intensity readouts of (a) Na^+ and (b) K^+ ions (100 mmol L^{-1}) as the concentrations of crown ether derivatives were varied from 3-50 μmol L^{-1}. Error bars represent standard error of the mean (n=3).

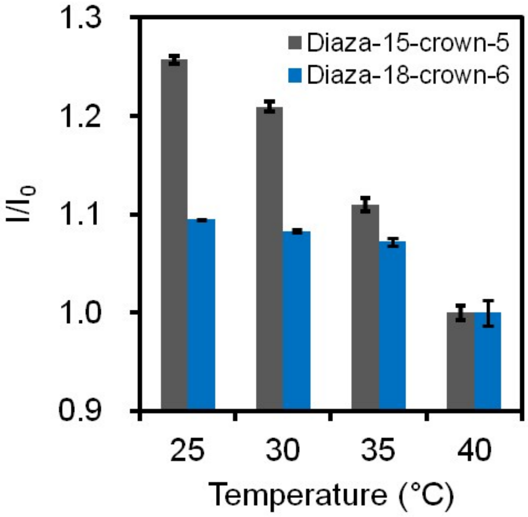

Figure S8. The effect of variation in temperature (25 to 40 °C) on fluorescence intensity at a constant ion concentration (100 mmol L^{-1}) and Diaza-15-crown-5 and Diaza-18-crown-6 concentration (25 µmol L^{-1}). Error bars represent standard error of the mean (n=3).

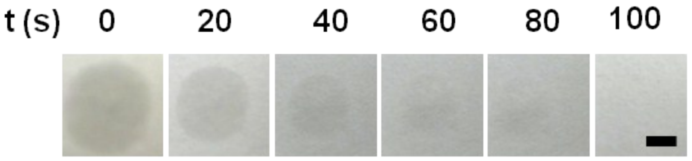

Figure S9. Photographs of DMSO (2 µL) on G1 filter paper dried in the air at 24°C. Scale bar=5 mm.

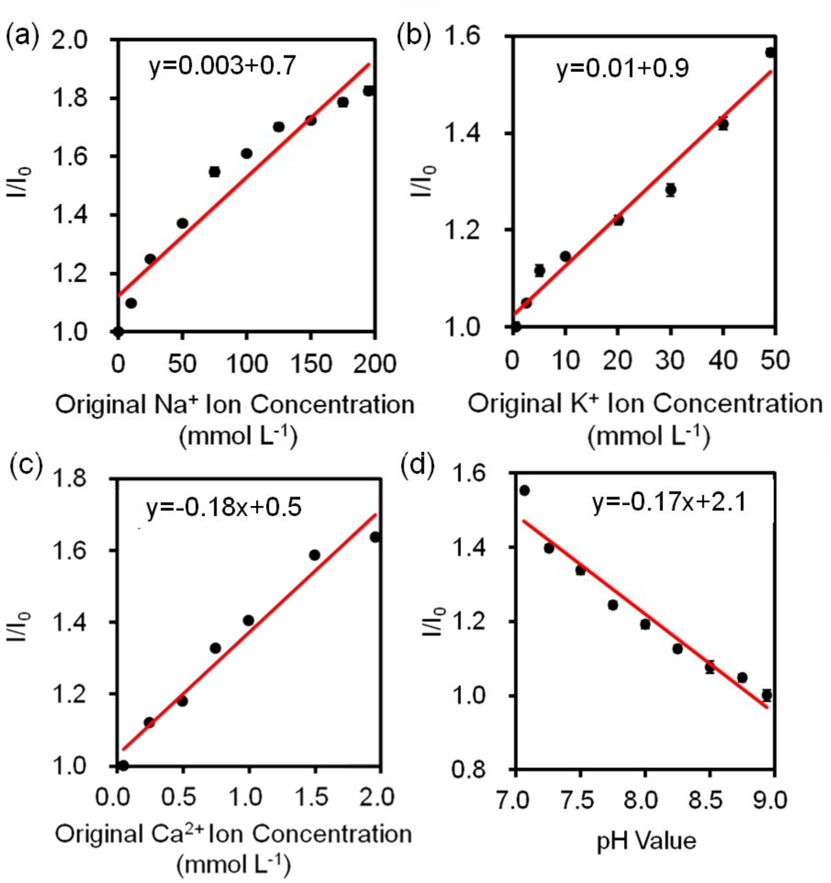

Figure S10. Calibration curves of electrolyte sensing on G1 paper using a microplate reader. (a) Na^+; (b) K^+; (c) Ca^{2+} ions and (d) pH. Error bars represent standard error of the mean (n=6).

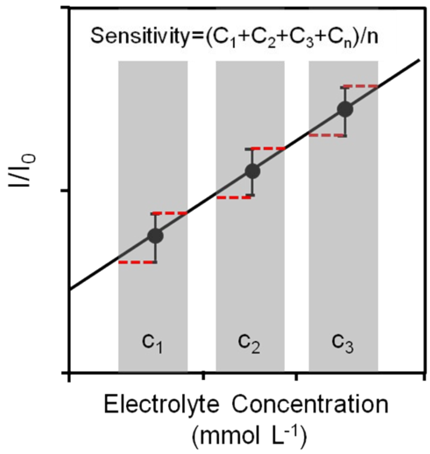

Figure S11. Calculation of sensitivity of the fluorescent sensors based on International Union of Pure and Applied Chemistry (IUPAC) guidelines.

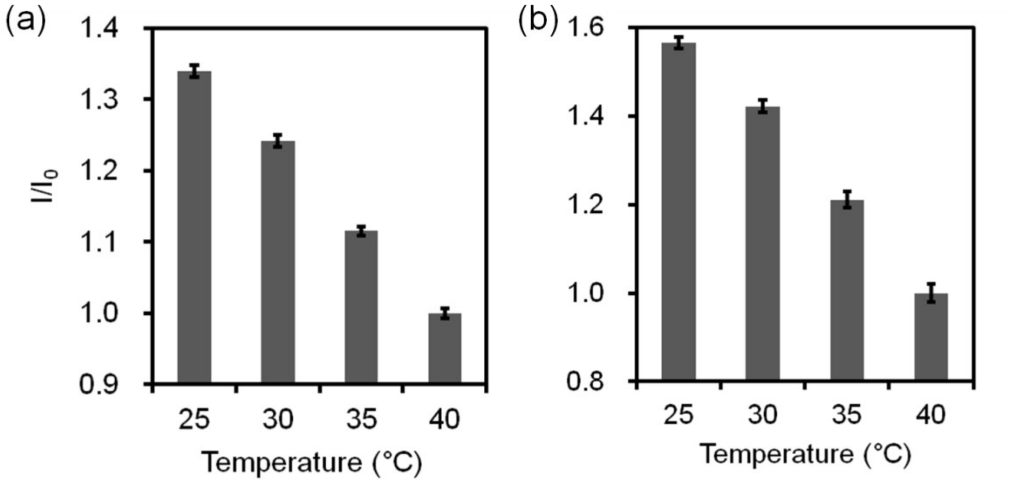

Figure S12. The effect of variation in temperature (25°C to 40 °C) on fluorescence intensity of (a) *o*-acetanisidide and (b) seminaphtorhodafluor (25 µmol L^{-1}) at a constant ion concentration (100 mmol L^{-1}). Error bars represent standard error of the mean (n=3).

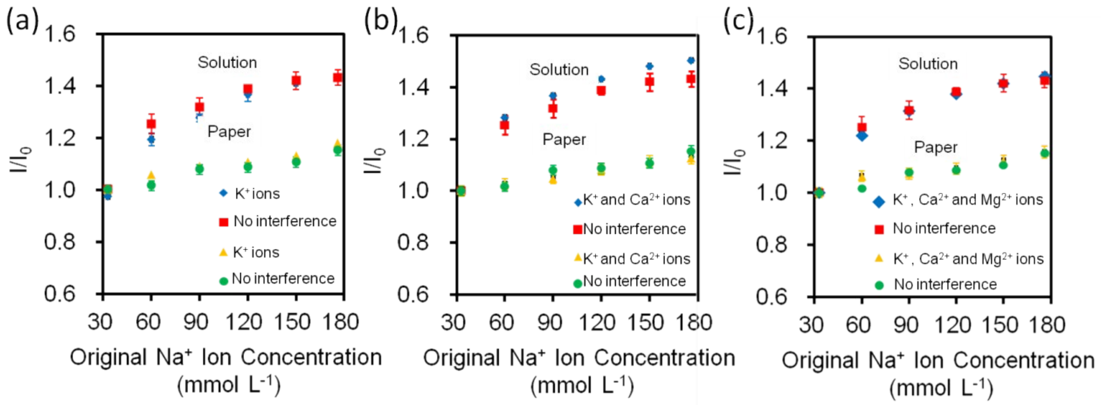

Figure S13. Quantifications of Na^+ ions interference both in buffer solution (Tris, pH 7.4, 150 mmol L^{-1}) and on G1 matrix after dilution at 24 °C. (a) Na^+ ions sensing in the presence of K^+ ions at the concentration of 42 mmol L^{-1}. (b) Na^+ ions sensing in the presence of K^+, Ca^{2+} at the concentrations of 42 mmol L^{-1} and 1.1 mmol L^{-1}, respectively. (c) Na^+ ions sensing in the presence of K^+, Ca^{2+} and Mg^{2+} at the concentrations of 42 mmol L^{-1}, 1.1 mmol L^{-1} and 0.9 mmol L^{-1}, respectively. Error bars represent standard error of the mean (n=3).

(a)

Component	Concentration
NaCl	125 mmol L^{-1}
KCl	20 mmol L^{-1}
$CaCl_2$	1 mmol L^{-1}
$MgCl_2$	0.5 mmol L^{-1}
Urea	5 mmol L^{-1}
NH_4Cl	3 mmol L^{-1}
Citric acid	31 μmol L^{-1}
Vitamin C	8 μmol L^{-1}
Albumins	3.94 g L^{-1}
Lysozyme	1.7 g L^{-1}
Tris-HCl	150 mmol L^{-1}
Tris-base	150 mmol L^{-1}

Adjust pH to 7.4

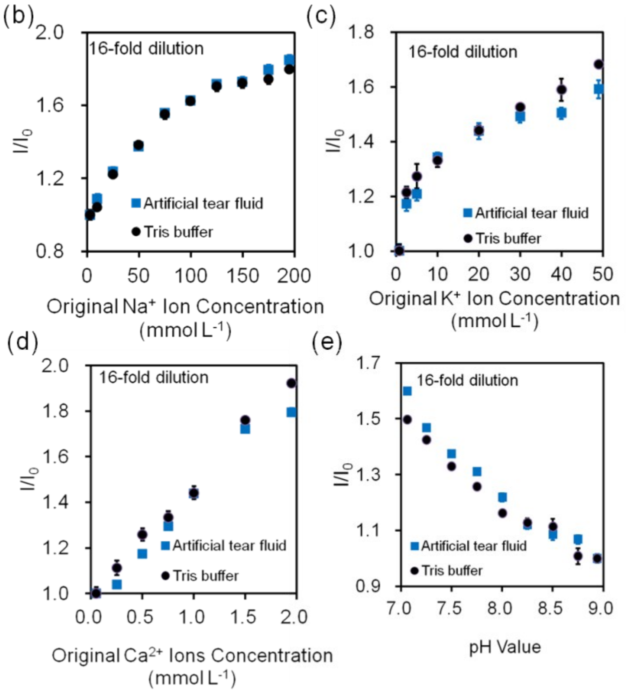

Figure S14. Quantification of ions and pH value in 16-fold diluted artificial tear fluid (pH=7.4) and Tris buffer (150 mmol L^{-1}) on G1 matrix at 24°C. (a) Composition of artificial tear fluid (pH=7.4); (b) Fluorescence Intensity readouts of Na^{+} ions in artificial tear fluid and Tris buffer were varied from ion-free solution to 200 mmol L^{-1} at a constant diaza-15-crown-5 concentration (25 μmol L^{-1}); (c) Fluorescence Intensity readout of K^{+} ions in artificial tear fluid and Tris buffer were varied from ion-free solution to 50 mmol L^{-1} at a constant diaza-18-crown-6 concentration (25 μmol L^{-1}); (d) Fluorescence Intensity readout of Ca^{2+} ions in artificial tear fluid and Tris buffer were varied from ion-free solution to 2.0 mmol L^{-1} at a constant o-acetanisidide concentration (25 μmol L^{-1}); (e) Fluorescence intensity readout of pH value in Tris buffer were varied from 7.0 to 9.0 at a constant seminaphtorhodafluor (25 μmol L^{-1}). Error bars represent standard error of the mean (n=3).

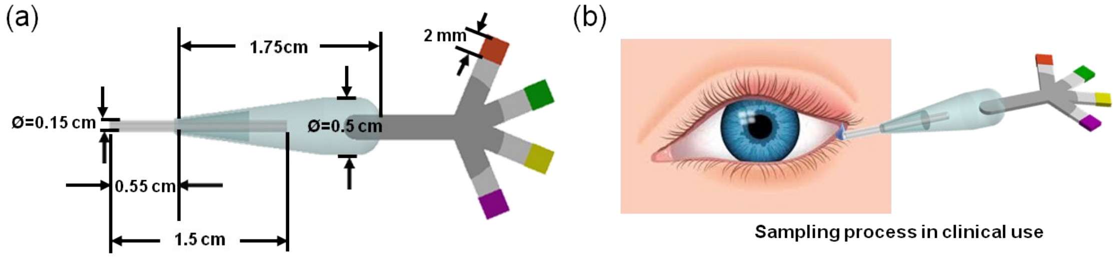

Figure S15. The design of the sample collection device and its potential clinical use. (a) The dimensions of the sample collection device. (b) Envisioned sampling process in clinical use.

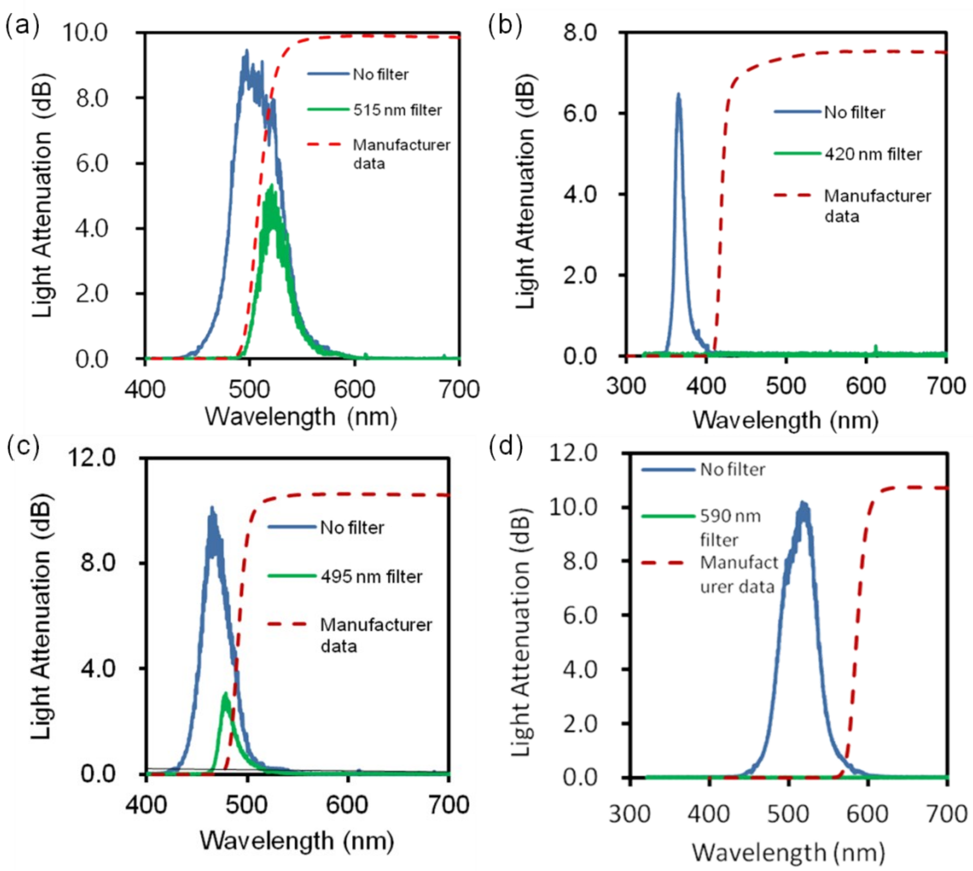

Figure S16. Light attenuation of LED lights using optical filters. (a) LED light at emission peak wavelength of 505 nm with 515 nm optical filter; (b) LED light at emission wavelength of 366 nm with 420 nm optical filter; (c) LED light at emission wavelength of 460 nm with 495 nm optical filter; (d) LED light at emission wavelength of 515 nm with 590 nm optical filter.

$$I_m = \frac{I_B + I_s}{I_B + I_o} \qquad \text{(Eq. S1)}$$

where I_m is the ratio for background noise subtraction, I_B is reflection through the filter; Is is the emission of the experiment sample; Io is the emission of the ion free sample.

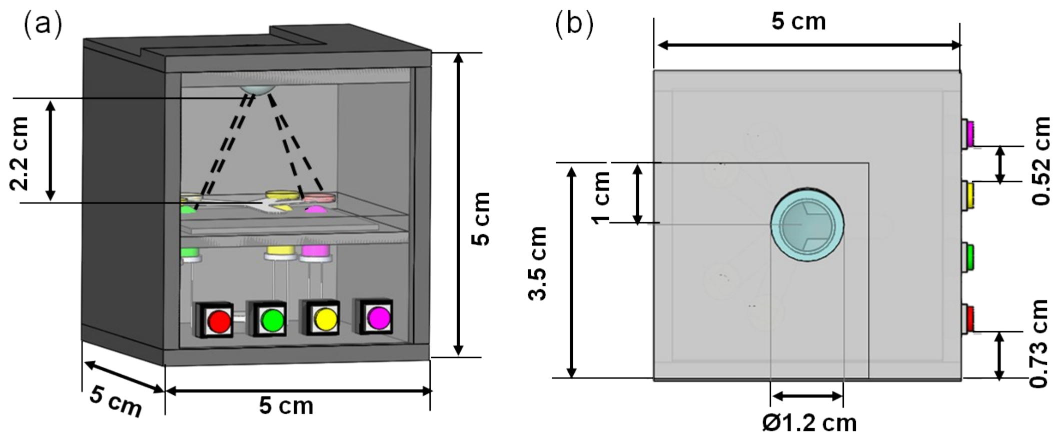

Figure S17. The dimensions of the portable readout device. (a) Side view; (b) Top view.

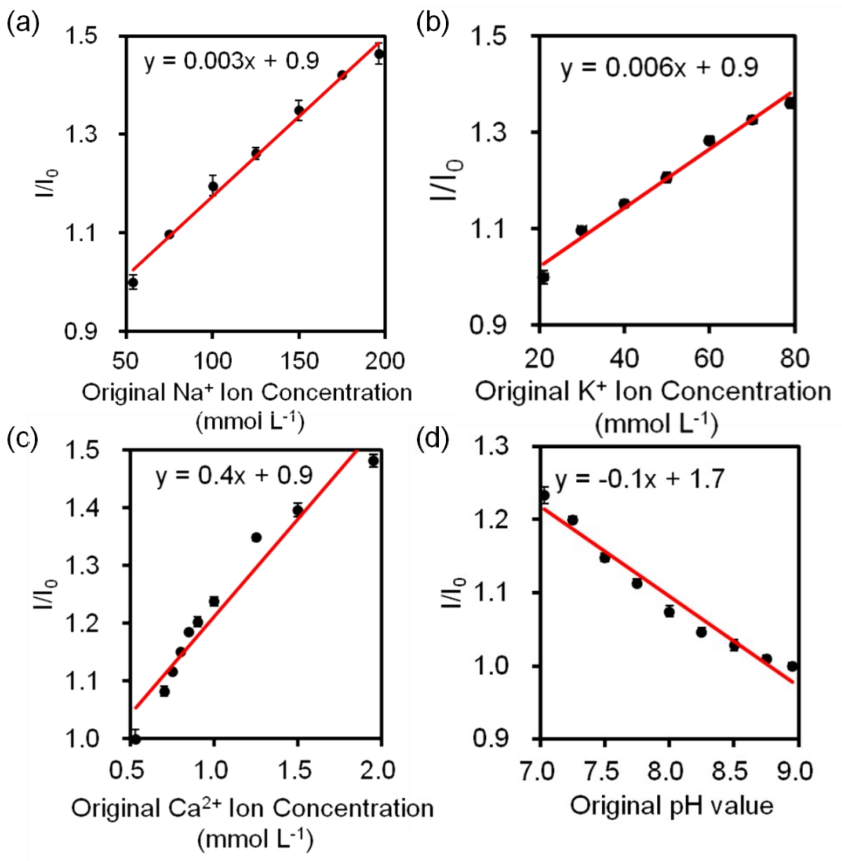

Figure S18. Calibration curves of electrolyte sensors using the paper-based microfluidic system: (a) Na^{+}, (b) K^{+}, (c) Ca^{2+} ions, and (d) pH. Error bars represent standard error of the mean (n=6).

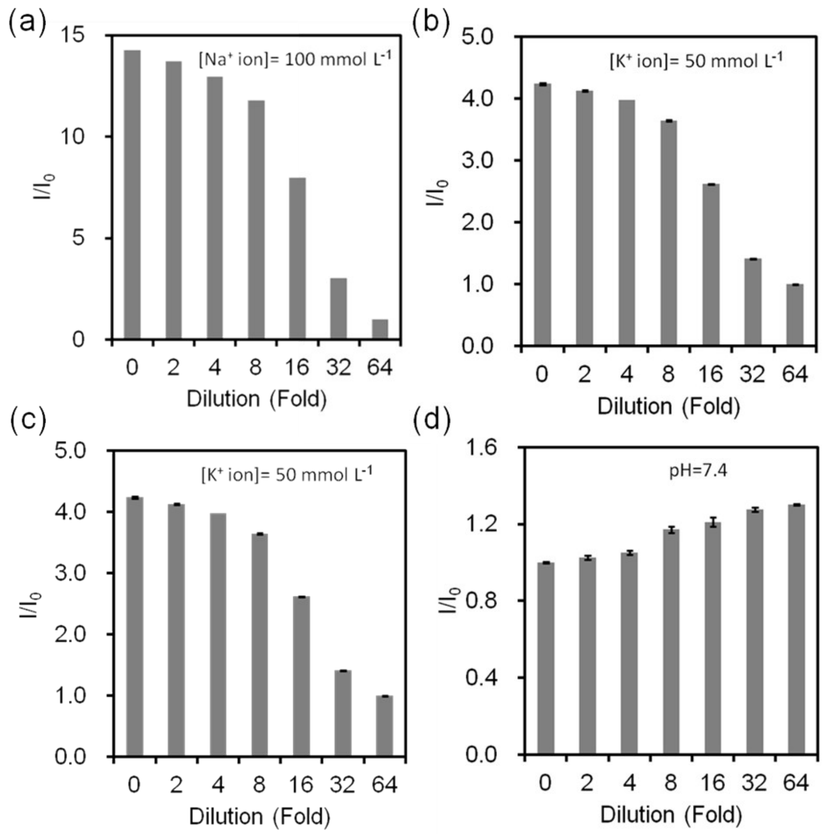

Figure S19. Measurement of sample dilution error in the quantification of electrolytes with different dilutions on G1 paper at 24 °C. (a) Na^+ ions solution (100 mmol L^{-1}); (b) K^+ ions solution (50 mmol L^{-1}); (c) Ca^{2+} ions (1 mmol L^{-1}); (d) pH (7.4). Error bars represent standard error of the mean (n=3).

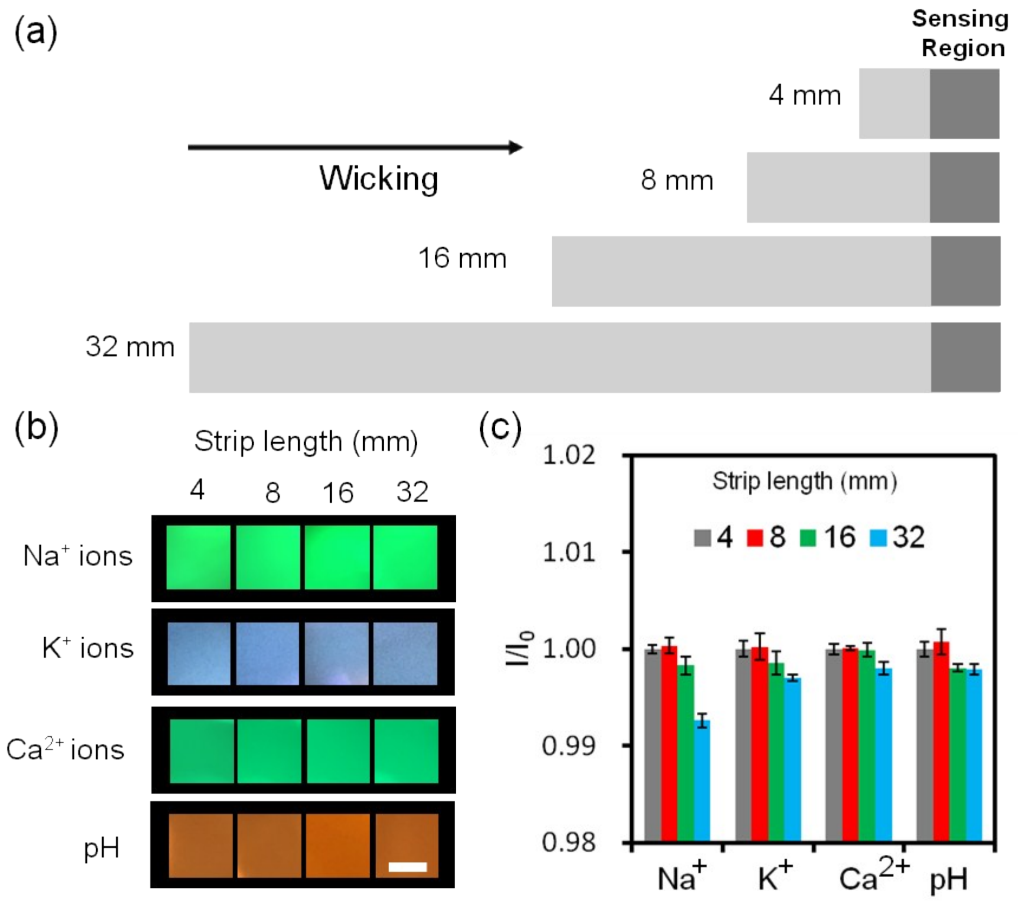

Figure S20. Quantifications of evaporation effect on fluorescence intensity measurements. (a) Electrolyte solution wicking on paper strips (width=2 mm) with different lengths (4, 8, 16, and 32 mm). (b) Na^{+} ion (100 mmol L^{-1}), K^{+} ion (50 mmol L^{-1}), Ca^{2+} ion (1 mmol L^{-1}) and pH (Tris solution, pH=7.4) sensing by a smartphone app. Scale bar=2 mm. (c) Electrolyte sensing readouts. Error bars represent standard error of the mean (n=3).

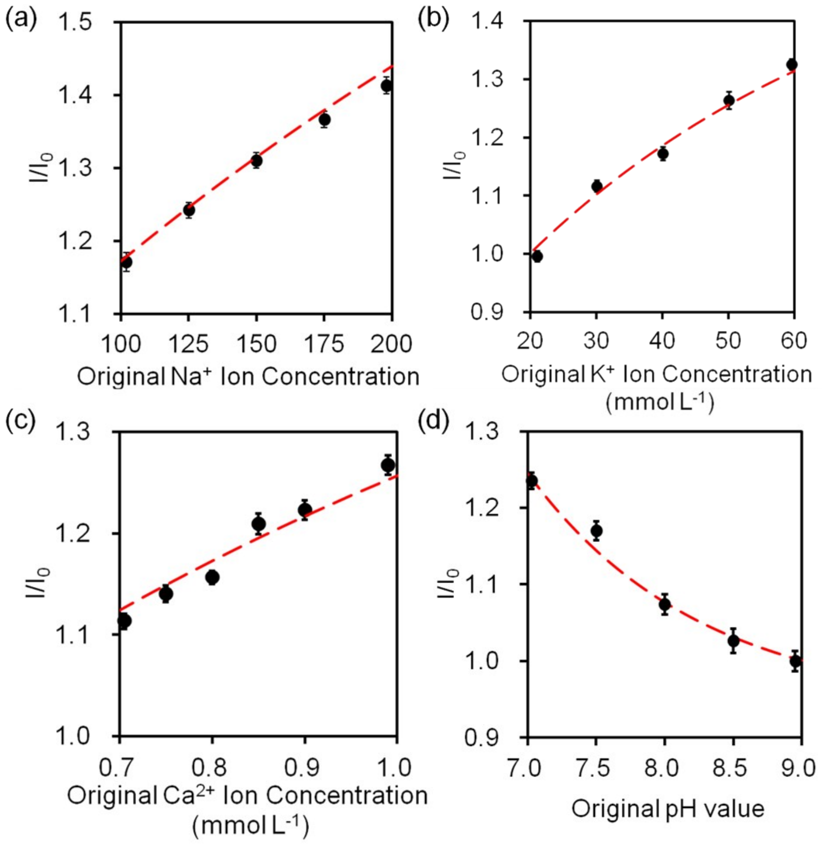

Figure S21. Quantification of electrolytes using paper-based microfluidic system in batch-to-batch experiments. (a) Na^{+} ions; (b) K^{+} ions; (c) Ca^{2+} ions, and (d) pH. Red dashes show fitted curves in Fig. 7. Error bars represent standard error of the mean (n=3).

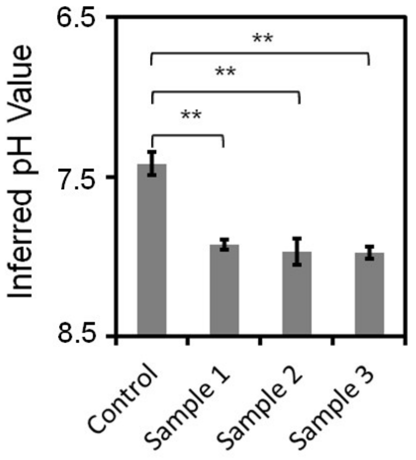

Figure S22. Quantitative analysis of simulated artificial tear samples (pH=7.4 as the control and pH=7.9 as the MGD and/or LGD sample) of inferred pH value in the mild stage. Error bars represent standard error of the mean (n=3).
